# Slater–Condon Rules and Spin–Orbit Couplings: 2‑(2-(2,5-Dimethoxybenzylidene)hydrazineyl)-4-(trifluoromethyl)thiazole a Test Case

**DOI:** 10.1021/acsomega.5c10359

**Published:** 2025-11-19

**Authors:** Hasnain Mehmood, Tashfeen Akhtar, Jesús Espinosa-Romero, Mauricio Maldonado-Domínguez, Jakub Višňák, Mirza Wasif Baig

**Affiliations:** † Department of Chemistry, 236246 Mirpur University of Science and Technology (MUST), Mirpur (AJK) 10250, Pakistan; ‡ Facultad de Química, Departamento de Química Orgánica, 7180 Universidad Nacional Autónoma de Mexico, Ciudad de Mexico 04510, Mexico; § Faculty of Mathematics and Physics, Charles University, Ke Karlovu 3, Prague 12116, Czech Republic; ∥ J. Heyrovsky Institute of Physical Chemistry of the Czech Academy of Sciences, Dolejskova 2155/3, Prague 18223, Czech Republic

## Abstract

Light-atom chromophores can display properties often associated with heavy-atom compounds, such as intersystem crossing, leading to phosphorescence and singlet oxygen generation, yet their use remains comparatively underexplored. Here, we report the synthesis of **HM610**, a derivative of the benzylidenehydrazinylthiazole light-atom chromophore backbone. Spin–orbit couplings (SOCs), computed with the sf-X2C–S-TDDFT method, follow Slater–Condon rules and predict moderate values. Trajectory surface hopping simulations further illustrate the role of dynamical effects in promoting ISC, yet these results together establish that **HM610** has only limited potential as a triplet sensitizer without further structural modification, such as heavy atom substitution. Based on the benchmarked (TD)­DFT protocol, a computational set studying six systematic analogues allowed us to study the influence of electron-donating (−OMe) and electron-withdrawing (−CF_3_) substituents on the common backbone, revealing the impact of substitution on the geometry and photophysics of light atom analogues of **HM610** and paving the way for future studies where the introduction of heavy atoms and their impact on triplet sensitization by this family of chromophores can be probed.

## Graphical abstract

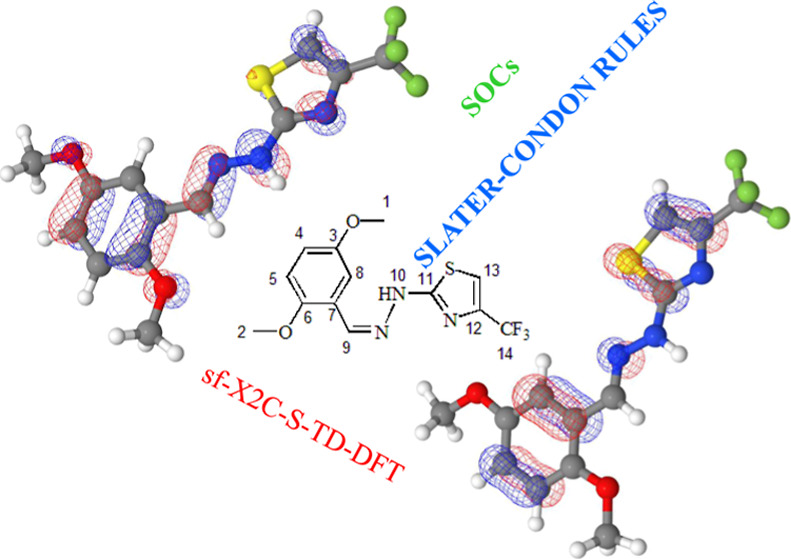

## Introduction

Thiazole derivatives represent a versatile class of heterocycles with broad relevance in medicinal chemistry and functional materials.^1–5^ Their electronic properties, dictated by the *N*,S-containing polarizable framework, make them attractive scaffolds for exploring photoinduced processes, where intersystem crossing (ISC) and spin–orbit coupling may strongly influence reactivity and application.^6,7^ Despite this importance, the excited-state dynamics of thiazole derivatives remain underexplored compared to those of other heteroaromatics, and their potential to serve as photon-to-chemistry energy transducers is mostly unknown.

Theoretical approaches, such as density functional theory (DFT),^8,9^ have been successful in computing the energetics of a broad range of molecular systems, from small metal clusters^10^ to medium-sized molecules,^11,12^ ultimately leading to complex condensed-phase molecular assemblies.^13^ Its time-dependent extension (TDDFT)^14^ provides practical tools for mapping electronic excitations,^15^ while higher-level correlated methods like coupled cluster singles and doubles (CC2) and the algebraic diagrammatic construction scheme through second order ADC(2) are increasingly used as benchmarks for validating functional performance in the description of excited states for single-reference molecules.^16–20^ Recent work shows that SOC can be appreciable even in molecules composed solely of light atoms,^7a,21^ raising questions about their role in ISC of organic chromophores. Spin–vibronic mechanism can also be crucial for inducing ISC in molecules with small SOCs.^22^ Trajectory surface hopping (TSH),^23,24^ which is a mixed quantum-classical method that is extensively used to simulate chemical reactions^24a,24b^ and excited-state dynamics,^24c,24d^ of photoactive molecules, can implicitly include first-order spin–vibronic effects.^22a^

In this study, we report the synthesis of 2-(2-(2,5-dimethoxybenzylidene)­hydrazinyl)-4-(trifluoromethyl)­thiazole (**HM610**), a derivative of the benzylidenehydrazinylthiazole organic backbone, and investigate its excited states through a combined experimental–computational strategy. Benchmarking multiple functionals against CC2 and ADC(2) allows us to assess the reliability of the TDDFT descriptions. The relativistic two-component sf-X2C-S-TDDFT method^25^ has been employed to explore SOCs between states of different multiplicities. TSH simulations have been employed to reveal the influence of dynamical effects on SOCs in this light-atom-based chromophore. TSH simulations for **HM610** evaluate the dependence of SOCs on nuclear coordinates. To place this molecule within a broader context, we constructed a systematic series of six analogues derived from the same thiazole–hydrazone–aryl backbone. The set spans different combinations of electron-donating (−OMe) and electron-withdrawing (−CF_3_) substituents at three defined positions, ranging from the unsubstituted parent to fully substituted variants, including **HM610**. Taken together, these results establish **HM610** and its analogues as reference systems for probing ISC in light-atom heterocycles, illustrating how donor–acceptor substitution patterns modulate their geometrical and photophysical landscape and allowing future comparisons with other light-atom and heavy-atom-containing chromophores.

## Experimental Methods and Materials

High-purity solvents and reagents were used in synthesis and were procured from well-reputed chemical suppliers (Sigma-Aldrich, Merck, and Fischer Scientific). Precoated aluminum sheets (Kiesel gel 60, F254, E. Merck, Germany) were used to monitor reaction progress via TLC. The UV–vis absorption spectrum was recorded on a Shimadzu Ultraviolet 1800 spectrophotometer using acetone as the solvent. Bruker OPUS ATR was used to record the IR spectrum. NMR experiments were performed on a Bruker DPX-400 MHz spectrometer. A Bruker Micro TOF-ESI mass spectrometer was used to record the mass spectrum.

### Synthesis of 2-(2-(2,5-Dimethoxybenzylidene)­hydrazineyl)-4-(trifluoromethyl)­thiazole (**HM610**)

An equimolar mixture of 2-(2,5-dimethoxybenzylidene)­hydrazine-1-carbothioamide (0.001 mol) and 3-bromo-1,1,1-trifluoroacetone (0.001 mol) was refluxed in ethanol for 4 h. The progress of the reaction and product purity was checked by TLC. Upon completion, the workup was done in ice cold water, which resulted in precipitation. The precipitates were washed with copious water, filtered, and dried. The UV spectrum was recorded in acetone, and the NMR spectrum was recorded in CDCl_3_.

### 2-(2-(2,5-Dimethoxybenzylidene)­hydrazineyl)-4-(trifluoromethyl)­thiazole (**HM610**)

Light yellow solid; Yield: 74%; *R*_f_: 0.90 (acetone/*n*-hexane, 1:1); λ_max_ = 329 nm; Fourier transform infrared (ATR) cm^–1^: 3178 (N–H stretching), 2958 (C–H aliphatic stretching), 1614 (thiazole skeletal vibrations), 1566 (C=N stretching), 1521, 1373, 1342, 1226 (C=C aromatic ring stretching), 1421 (C–H aliphatic bending), 1110 (C–O stretching), 1008–704 (characteristic thiazole vibrations); ^1^H NMR (400 MHz, CDCl_3_): δ (ppm) 10.26 (s, 1H, H–N-), 8.27 (s, 1H, H–C=N-), 7.49 (d, 1H, Ar–H, *J* = 3.0 Hz), 7.14 (s 1H, thiazole-H), 6.94 (dd, 1H, Ar–H, *J* = 9.0, 3.0 Hz), 6.87 (d, 1H, Ar–H, *J* = 9.0 Hz) 3.86 (s, 3H, –OCH_3_), 3.85 (s, 3H, –OCH_3_); ^19^F NMR (375 MHz): δ −64.65 (−CF_3_); ^13^C NMR (100 MHz): δ 170.7 (thiazole C2), 153.7 (Ar–C–OMe), 152.5 (Ar–C–OMe), 140.7 (q,^2^ *J*_C‑CF_3__ = 36.7 Hz, thiazole C4), 139.4 (-C=N-), 122.4 (q, ^1^ *J*_C–F_ = 271 Hz, –CF_3_), 119.2, 117.2 (Ar–C), 112.2 (q, ^3^ *J*_C–CF_3__ = 4.0 Hz, thiazole C5), 110.3 (Ar–C), 56.2 (−OCH_3_), 55.8 (−OCH_3_); HRMS: *m*/*z* calculated for C_13_H_12_F_3_N_3_O_2_S, [M + H]^+^: 332.0681, found: 332.0674, [M + Na]^+^: calculated: 354.0500, found: 354.0491, [2M + Na]^+^: calculated: 685.1102, found: 685.1114.

### Computational Methodology

All quantum chemical calculations were performed using TURBOMOLE 7.2.1,^26^ BDF,^27^ and Gaussian 09.^28^ Excited-state dynamics were simulated with Newton-X.^29^

#### Geometry Optimization

Ground-state geometries of all studied thiazole derivatives were optimized with the meta-hybrid functional M062X^30^ and the def2-TZVP basis set.^31^ Optimizations were followed by vibrational frequency analyses to confirm that the structures corresponded to true minima on the potential energy surface, verified by the absence of imaginary frequencies. Calculations employed an ultrafine integration grid (int = ultrafine), were carried out in the gas phase without explicit solvent effects, and used reduced symmetry conditions (nosymm) to avoid artificial restrictions. Grimme’s D3 dispersion correction was included in all DFT optimizations.^32^

#### Excited-State Calculations

Vertical excitation energies were obtained with correlated wave function methods [CC2 and ADC(2)] using the RI approximation in TURBOMOLE.^33^ In parallel, TDDFT calculations were performed with six functionals (M062X, BHLYP,^34^ CAM-B3LYP,^35^ ωB97, ωB97X, and ωB97X-D^36^) and the def2-TZVP basis set. For each compound, 20 singlet excited states were computed on both singlet and triplet optimized geometries, enabling characterization of excitation energies, oscillator strengths, natural transition orbitals, and singlet–triplet gaps. Using a consistent methodological scheme ensured a direct comparison between ground- and excited-state properties. Solvent effects on excitation energies were also explored with the polarizable continuum model (PCM).^37^

#### Spin–Orbit Couplings

Spin–orbit couplings (SOCs) were evaluated with the sf-X2C–S-TDDFT approach as implemented in BDF.^38^ This method incorporates a Douglas–Kroll–Hess (DKH1)-like spin–orbit Hamiltonian, with one-electron terms included explicitly and two-electron contributions treated via the molecular mean-field approximation. The CIS-like wave function is constructed from TDDFT excitation vectors (X + Y) with normalization, providing a reliable treatment of relativistic effects in molecules containing light to medium atoms.^39^

#### DMRG Calculations

DMRG calculations have been done using the program MOLMPS^40^ employing the def-TZVP basis set to evaluate multireference character^41^ and orbital entropies^42^ in **HM610**. DMRG calculations have been used in recent years to evaluate the multireference character of different molecular systems.^43^ DMRG calculations have been performed in the active space of 20 electrons in 20 orbitals around the HOMO, and the initial DFT orbitals were split-localized.

#### Surface Hopping Molecular Dynamics

Excited-state dynamics were simulated with Newton-X. The nuclear ensemble approach (NEA) was applied to simulate absorption spectra,^44^ while mixed quantum–classical trajectory surface hopping (Tully’s FSSH) was employed for nonadiabatic dynamics, including intersystem crossing.^45,46^ Both time-derivative couplings and SOCs were included by using a three-step integrator scheme. The Tamm–Dancoff approximation (TDA) was used within TDDFT to minimize triplet instabilities^47,48^ and improve the description of conical intersections.^49^ Dynamics trajectories were propagated at the BHLYP-D3/dhf-TZVP level of theory as employed in our previous study.^6b^

#### NMR Chemical Shifts

^1^H and ^13^C chemical shifts were calculated with the gauge-including atomic orbital (GIAO) method, using reference TMS shielding constants at the same theoretical level.^50^ Calculations were carried out at B3LYP-D3/cc-pVQZ both in the gas phase and in CDCl_3_ (PCM). Additional calculations with M062X/cc-pVQZ and M062X/def2-QZVPP (PCM, Gaussian) were performed for comparison.^51^ The use of B3LYP for NMR predictions is supported by previous reports of its accuracy.^52^

## Results and Discussion

### Synthesis of 2-(2-(2,5-Dimethoxybenzylidene)­hydrazineyl)-4-(trifluoromethyl)­thiazole (3)

The synthesis of 2-(2-(2,5-dimethoxybenzylidene)­hydrazineyl)-4-(trifluoromethyl)­thiazole was achieved by the literature-reported method,^53^ as shown in Scheme 1. An equimolar mixture of 2-(2,5-dimethoxybenzylidene)­hydrazine-1-carbothioamide and 3-bromo-1,1,1-trifluoroacetone was refluxed in ethanol for 4 h.

**Scheme 1:**
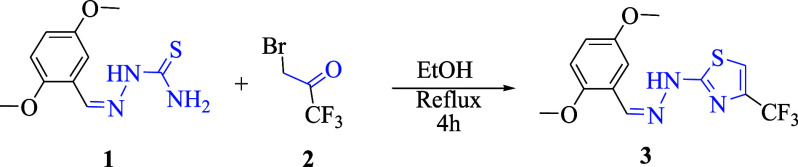
Synthesis of 2-(2-(2,5-dimethoxybenzylidene)­hydrazineyl)-4-(trifluoromethyl)-thiazole

The synthesized compound was initially characterized by UV–vis and IR spectroscopy. The structure of the synthesized compound was established through NMR spectroscopy. The hydrazone functionality is indicated by characteristic amine 3178 cm^–1^ stretching. This connectivity was further supported by a characteristic (azomethine) one-proton singlet at 8.27 ppm in the ^1^H NMR spectrum. ^13^C NMR spectroscopy added confirmation to the presence of an azomethine linkage by a signal at 139.4 ppm. Similarly, thiazole moiety was indicated by characteristic thiazole vibrations^54^ in the range of 1008–704 cm^–1^ and further confirmed by ^13^C NMR, where the thiazole C2 signal was observed at 170.7 ppm, thiazole C4 at 140.7 ppm, and thiazole C5 at 112.2 ppm. The aromatic protons and carbons appeared within the usually reported ranges.

### Benchmarking of Density Functionals

Coupled-cluster-based wave function methods, such as CC2 and ADC(2), have been shown to yield comparable band gap energies across a wide range of organic dyes^19^ and provide reliable estimates for excited-state decay rate constants.^55^ In this study, these methods are employed to benchmark the TDDFT functionals under investigation. For the **HM610** molecule, all vertical excitation energies computed using wave function methods (CC2 and ADC(2)) and TDDFT functionals (M062X; ωB97; ωB97X; ωB97X-D; BHLYP; and CAM-B3LYP) are summarized in Tables 1 and 2. At first glance, the data suggest that M062X yields excitation energies more closely aligned with those from CC2 and ADC(2) than other functionals, in agreement with previous findings.^55^ Vertical excitation energies of the most important states are tabulated in Tables S1–S3. A more detailed comparison is provided in Tables S4–S6 which show the differences in excitation energies across the various methods. The maximum absolute energy difference between ADC(2) and CC2 for the first seven singlet excited states is 0.05 eV, while the energies for the first ten triplet states are identical, an agreement consistent with trends reported for other organic molecules.^19^

**Table 1:** Vertical Excitation Energies Computed with Four Different Electronic Structure Methods at the S_0_ Optimized Geometry

states	CC(2)	character	ADC(2)	character	M062X	character	ωB97	character
S_1_	3.79 (0.50)	( ${\pi ;\pi^{*})}^{1}$	3.75 (0.46)	( ${\pi ;\pi^{*})}^{1}$	3.96 (0.66)	( ${\pi ;\pi^{*})}^{1}$	4.17 (0.61)	( ${\pi ;\pi^{*})}^{1}$
S_2_	4.21 (0.33)	( ${\pi ;\pi^{*})}^{1}$	4.16 (0.24)	( ${\pi ;\pi^{*})}^{1}$	4.40 (0.09)	( ${\pi ;\pi^{*})}^{1}$	4.62 (0.16)	( ${\pi ;\pi^{*})}^{1}$
S_3_	5.41 (0.09)	( ${\pi ;\pi^{*})}^{1}$	5.38 (0.12)	( ${\pi ;\pi^{*})}^{1}$	5.43 (0.00)	( ${\pi ;\sigma^{*})}^{1}$	5.60 (0.03)	( ${\pi ;\pi^{*})}^{1}$
S_4_	5.44 (0.02)	( ${\pi ;\sigma^{*})}^{1}$	5.47 (0.00)	( ${\pi ;\sigma^{*})}^{1}$	5.46 (0.11)	( ${\pi ;\pi^{*})}^{1}$	5.85 (0.00)	( ${\pi ;\sigma^{*})}^{1}$
S_5_	5.58 (0.03)	( ${\pi ;\pi^{*})}^{1}$	5.53 (0.04)	( ${\pi ;\pi^{*})}^{1}$	5.52 (0.00)	( ${\sigma ;\pi^{*})}^{1}$	5.87 (0.00)	( ${\sigma ;\pi^{*})}^{1}$
S_6_	5.75 (0.00)	( ${\sigma ;\pi^{*})}^{1}$	5.70 (0.00)	( ${\sigma ;\pi^{*})}^{1}$	5.60 (0.00)	( ${\pi ;\pi^{*})}^{1}$	5.98 (0.36)	( ${\pi ;\pi^{*})}^{1}$
S_7_	5.87 (0.19)	( ${\pi ;\pi^{*})}^{1}$	5.86 (0.21)	( ${\pi ;\pi^{*})}^{1}$	5.95 (0.04)	( ${\pi ;\sigma^{*})}^{1}$	6.39 (0.01)	( ${\pi ;\pi^{*})}^{1}$
T_1_	2.99 (0.00)	${\left(\pi ;\pi^{*}\right)}^{3}$	2.99 (0.00)	${\left(\pi ;\pi^{*}\right)}^{3}$	2.80 (0.00)	${\left(\pi ;\pi^{*}\right)}^{3}$	2.40 (0.00)	${\left(\pi ;\pi^{*}\right)}^{3}$
T_2_	3.62 (0.00)	${\left(\pi ;\pi^{*}\right)}^{3}$	3.62 (0.00)	${\left(\pi ;\pi^{*}\right)}^{3}$	3.53 (0.00)	${\left(\pi ;\pi^{*}\right)}^{3}$	3.39 (0.00)	${\left(\pi ;\pi^{*}\right)}^{3}$
T_3_	4.00 (0.00)	${\left(\pi ;\pi^{*}\right)}^{3}$	4.00 (0.00)	${\left(\pi ;\pi^{*}\right)}^{3}$	3.86 (0.00)	${\left(\pi ;\pi^{*}\right)}^{3}$	3.46 (0.00)	${\left(\pi ;\pi^{*}\right)}^{3}$
T_4_	4.42 (0.00)	${\left(\pi ;\pi^{*}\right)}^{3}$	4.42 (0.00)	${\left(\pi ;\pi^{*}\right)}^{3}$	4.31 (0.00)	${\left(\pi ;\pi^{*}\right)}^{3}$	3.87 (0.00)	${\left(\pi ;\pi^{*}\right)}^{3}$
T_5_	4.81(0.00)	${\left(\pi ;\pi^{*}\right)}^{3}$	4.81(0.00)	${\left(\pi ;\pi^{*}\right)}^{3}$	4.71 (0.00)	${\left(\pi ;\pi^{*}\right)}^{3}$	4.61 (0.00)	${\left(\pi ;\pi^{*}\right)}^{3}$
T_6_	5.21 (0.00)	( ${\sigma ;\pi^{*})}^{3}$	5.21 (0.00)	( ${\sigma ;\pi^{*})}^{3}$	4.90 (0.00)	( ${\sigma ;\pi^{*})}^{3}$	4.99 (0.00)	$${\left(\pi ;\pi^{*}\right)}^{3}$$
T_7_	5.28 (0.00)	$${\left(\pi ;\sigma^{*}\right)}^{3}$$	5.28 (0.00)	$${\left(\pi ;\pi^{*}\right)}^{3}$$	5.13 (0.00)	$${\left(\pi ;\pi^{*}\right)}^{3}$$	5.10 (0.00)	( ${\sigma ;\pi^{*})}^{3}$
T_8_	5.32 (0.00)	${\left(\pi ;\pi^{*}\right)}^{3}$	5.32 (0.00)	${\left(\pi ;\sigma^{*}\right)}^{3}$	5.25 (0.00)	${\left(\pi ;\sigma^{*}\right)}^{3}$	5.51 (0.00)	${\left(\pi ;\sigma^{*}\right)}^{3}$
T_9_	5.72 (0.00)	${\left(\pi ;\pi^{*}\right)}^{3}$	5.72 (0.00)	${\left(\pi ;\pi^{*}\right)}^{3}$	5.47 (0.00)	${\left(\pi ;\pi^{*}\right)}^{3}$	5.68 (0.00)	${\left(\pi ;\pi^{*}\right)}^{3}$
T_10_	5.89 (0.00)	${\left(\pi ;\pi^{*}\right)}^{3}$	5.89 (0.00)	${\left(\pi ;\pi^{*}\right)}^{3}$	5.63 (0.00)	${\left(\pi ;\pi^{*}\right)}^{3}$	5.81 (0.00)	${\left(\pi ;\pi^{*}\right)}^{3}$

**Table 2:** Vertical Excitation Energies Computed with Four Different Electronic Structure Methods at the S_0_ Optimized Geometry

states	ωB97X	character	ωB97X-D	character	CAM-B3LYP	character	BHLYP	character
S_1_	4.09 (0.62)	( ${\pi ;\pi^{*})}^{1}$	3.93 (0.61)	( ${\pi ;\pi^{*})}^{1}$	3.91 (0.61)	( ${\pi ;\pi^{*})}^{1}$	4.06 (0.68)	( ${\pi ;\pi^{*})}^{1}$
S_2_	4.54 (0.15)	( ${\pi ;\pi^{*})}^{1}$	4.37 (0.16)	( ${\pi ;\pi^{*})}^{1}$	4.33 (0.14)	( ${\pi ;\pi^{*})}^{1}$	4.49 (0.07)	( ${\pi ;\pi^{*})}^{1}$
S_3_	5.55 (0.05)	( ${\pi ;\pi^{*})}^{1}$	5.44 (0.06)	( ${\pi ;\pi^{*})}^{1}$	5.42 (0.07)	( ${\pi ;\pi^{*})}^{1}$	5.50 (0.08)	( ${\pi ;\pi^{*})}^{1}$
S_4_	5.70 (0.00)	( ${\pi ;\sigma^{*})}^{1}$	5.48 (0.00)	( ${\pi ;\sigma^{*})}^{1}$	5.45 (0.00)	( ${\pi ;\sigma^{*})}^{1}$	5.60 (0.00)	( ${\pi ;\sigma^{*})}^{1}$
S_5_	5.79 (0.00)	( ${\sigma ;\pi^{*})}^{1}$	5.65 (0.12)	( ${\pi ;\pi^{*})}^{1}$	5.55 (0.08)	( ${\pi ;\pi^{*})}^{1}$	5.65 (0.06)	( ${\pi ;\pi^{*})}^{1}$
S_6_	5.89 (0.28)	( ${\pi ;\pi^{*})}^{1}$	5.66 (0.00)	( ${\sigma ;\pi^{*})}^{1}$	5.67 (0.00)	( ${\sigma ;\pi^{*})}^{1}$	5.90 (0.00)	( ${\sigma ;\pi^{*})}^{1}$
S_7_	6.25 (0.13)	( ${\pi ;\pi^{*})}^{1}$	5.98 (0.18)	( ${\pi ;\pi^{*})}^{1}$	5.90 (0.20)	( ${\pi ;\pi^{*})}^{1}$	6.06 (0.21)	( ${\pi ;\pi^{*})}^{1}$
T_1_	2.44 (0.00)	${\left(\pi ;\pi^{*}\right)}^{3}$	2.50 (0.00)	${\left(\pi ;\pi^{*}\right)}^{3}$	2.38 (0.00)	${\left(\pi ;\pi^{*}\right)}^{3}$	2.06 (0.00)	${\left(\pi ;\pi^{*}\right)}^{3}$
T_2_	3.39 (0.00)	${\left(\pi ;\pi^{*}\right)}^{3}$	3.34 (0.00)	${\left(\pi ;\pi^{*}\right)}^{3}$	3.29 (0.00)	${\left(\pi ;\pi^{*}\right)}^{3}$	3.16 (0.00)	${\left(\pi ;\pi^{*}\right)}^{3}$
T_3_	3.46 (0.00)	${\left(\pi ;\pi^{*}\right)}^{3}$	3.51 (0.00)	${\left(\pi ;\pi^{*}\right)}^{3}$	3.40 (0.00)	${\left(\pi ;\pi^{*}\right)}^{3}$	3.36 (0.00)	${\left(\pi ;\pi^{*}\right)}^{3}$
T_4_	3.92 (0.00)	${\left(\pi ;\pi^{*}\right)}^{3}$	3.99 (0.00)	${\left(\pi ;\pi^{*}\right)}^{3}$	3.86 (0.00)	${\left(\pi ;\pi^{*}\right)}^{3}$	3.67 (0.00)	${\left(\pi ;\pi^{*}\right)}^{3}$
T_5_	4.57 (0.00)	${\left(\pi ;\pi^{*}\right)}^{3}$	4.52 (0.00)	${\left(\pi ;\pi^{*}\right)}^{3}$	4.46 (0.00)	${\left(\pi ;\pi^{*}\right)}^{3}$	4.49 (0.00)	${\left(\pi ;\pi^{*}\right)}^{3}$
T_6_	4.95 (0.00)	${\left(\pi ;\pi^{*}\right)}^{3}$	4.91 (0.00)	${\left(\pi ;\pi^{*}\right)}^{3}$	4.83 (0.00)	${\left(\pi ;\pi^{*}\right)}^{3}$	4.73 (0.00)	${\left(\pi ;\pi^{*}\right)}^{3}$
T_7_	5.01 (0.00)	( ${\sigma ;\pi^{*})}^{3}$	4.92 (0.00)	( ${\sigma ;\pi^{*})}^{3}$	4.87 (0.00)	( ${\sigma ;\pi^{*})}^{3}$	4.98 (0.00)	( ${\sigma ;\pi^{*})}^{3}$
T_8_	5.40 (0.00)	( ${\pi ;\sigma^{*})}^{3}$	5.22 (0.00)	$${\left(\pi ;\sigma^{*}\right)}^{3}$$	5.17 (0.00)	$${\left(\pi ;\sigma^{*}\right)}^{3}$$	5.27 (0.00)	$${\left(\pi ;\sigma^{*}\right)}^{3}$$
T_9_	5.60 (0.00)	${\left(\pi ;\pi^{*}\right)}^{3}$	5.46 (0.00)	${\left(\pi ;\pi^{*}\right)}^{3}$	5.36 (0.00)	${\left(\pi ;\pi^{*}\right)}^{3}$	5.42 (0.00)	${\left(\pi ;\pi^{*}\right)}^{3}$
T_10_	5.72 (0.00)	${\left(\pi ;\pi^{*}\right)}^{3}$	5.58 (0.00)	${\left(\pi ;\pi^{*}\right)}^{3}$	5.49 (0.00)	${\left(\pi ;\pi^{*}\right)}^{3}$	5.62 (0.00)	${\left(\pi ;\pi^{*}\right)}^{3}$

From the comparison of six functionals (M062X, ωB97, ωB97X, ωB97X-D, CAM-B3LYP, and BHLYP) against the CC2/ADC(2) benchmarks, clear trends emerge. For the first excited state (S_1_), M062X and CAM-B3LYP give the closest agreement, with CAM-B3LYP slightly outperforming all others. Across the entire set of singlet and triplet states, however, ωB97X/ωB97X-D and M062X show the most balanced accuracy, while BHLYP and ωB97 tend to overestimate deviations. CAM-B3LYP performs very well for S_1_ but displays larger systematic errors for triplets. Overall, CAM-B3LYP is best if S_1_ is the priority, while M062X and ωB97X/ωB97X-D are more reliable for a balanced description of the full excited-state manifold. Nonetheless, the electronic state characters of the first two singlets, the first five triplets, and the two highest triplet states remain consistent across all methods.

Notably, some differences in the excited-state character are observed. For singlet states, the character of S_3_ and S_4_ is swapped between ${\left(\pi ;\sigma^{*}\right)}^{1}$ and ${\left(\pi ;\pi^{*}\right)}^{1}$ configurations for M062X relative to CC2 and ADC(2). Similarly, for S_5_ and S_6_, the character is reversed between ( ${\sigma ;\pi^{*})}^{1}$ and ${\left(\pi ;\pi^{*}\right)}^{1}$ configurations in both M062X and ωB97 compared to CC2 and ADC(2). Among the triplets, T_7_ and T_8_ exhibit a character swap between ${\left(\pi ;\sigma^{*}\right)}^{3}$ and ${\left(\pi ;\pi^{*}\right)}^{3}$ configurations in ADC(2) and M062X relative to CC2. A similar swap occurs for T_6_ and T_7_ between ( ${\sigma ;\pi^{*})}^{3}$ and ${\left(\pi ;\pi^{*}\right)}^{3}$ configurations in ωB97 compared to ADC(2). For the singlet states, ωB97X predicts the same ordering as CC2 and ADC(2) for S_3_ and S_4_, both retaining the ${\left(\pi ;\pi^{*}\right)}^{1}$ and ${\left(\pi ;\sigma^{*}\right)}^{1}$ characters, respectively. The S_5_ and S_6_ states are also consistent with CC2/ADC(2), where S_5_ is ( ${\sigma ;\pi^{*})}^{1}$ and S_6_ is ${\left(\pi ;\pi^{*}\right)}^{1}$ , so no character swaps occur among the singlets relative to the reference methods. In contrast, ωB97X-D, CAM-B3LYP, and BHLYP retain the CC2/ADC(2) ordering for S_3_ and S_4_, but they exhibit a reversal for S_5_ and S_6_: these functionals assign S_5_ as ${\left(\pi ;\pi^{*}\right)}^{1}$ and S_6_ as ( ${\sigma ;\pi^{*})}^{1}$ , opposite to the character distribution found with CC2/ADC(2). Among the triplets, ωB97X differs from CC2/ADC(2) by assigning T_6_ as ${\left(\pi ;\pi^{*}\right)}^{3}$ and T_7_ as ( ${\sigma ;\pi^{*})}^{3}$ , thereby reversing the ( ${\sigma ;\pi^{*})}^{3}$ / ${\left(\pi ;\pi^{*}\right)}^{3}$ ordering seen with CC2/ADC(2). The same T_6_/T_7_ reversal is also observed with ωB97X-D, CAM-B3LYP, and BHLYP, which assign T_6_ as ${\left(\pi ;\pi^{*}\right)}^{3}$ and T_7_ as ( ${\sigma ;\pi^{*})}^{3}$ instead of the CC2/ADC(2) pattern. Furthermore, these three functionals predict a distinct rearrangement of T_7_ and T_8_ compared to both CC2 and ADC(2); rather than alternating ${\left(\pi ;\sigma^{*}\right)}^{3}$ and ${\left(\pi ;\pi^{*}\right)}^{3}$ as in CC2, or ${\left(\pi ;\pi^{*}\right)}^{3}$ and ${\left(\pi ;\sigma^{*}\right)}^{3}$ as in ADC(2)/M062X, they place T_7_ as ( ${\sigma ;\pi^{*})}^{3}$ and T_8_ as ${\left(\pi ;\sigma^{*}\right)}^{3}$ . As mentioned above, M062X and CAM-B3LYP show the closest agreement with high-level wave function methods.

Based on the presented results, the effect of solvent on vertical excitation energies was evaluated using only the M062X and CAM-B3LYP functionals for the **HM610** molecule, employing the PCM solvation model with the dielectric constant of dimethyl sulfoxide (DMSO). The vertical excitation energies of **HM610** presented in Table S7 show minimal differences between the gas phase and DMSO, with most shifts within ±0.05 eV. As shown in Table S8, both M062X and CAM-B3LYP functionals maintain consistent excitation character across environments with only a few state-specific changes (e.g., S_3_, S_4_). These results indicate that solvent effects on the excited-state properties of **HM610** are relatively minor.

The simulated absorption spectra of **HM610** in the gas phase were calculated at the TDDFT/BHLYP-D3/dhf-TZVP level of theory by using the nuclear ensemble approach (NEA) presented in Figure 1. In NEA, photoabsorption cross sections are averaged over configuration-space points, either sampled from phase-space distributions or collected during a ground-state MD simulation. The cross-section for each geometry depends on the sum of oscillator strengths of transitions, combined with Gaussian line shapes centered at their respective vertical excitation energies. The calculations used nuclear configurations sampled from the Wigner distribution of a canonical ensemble of quantum harmonic oscillators at 298 K. Seven of the lowest singlet excited states of **HM610** were included in the simulation.

**Figure 1:**
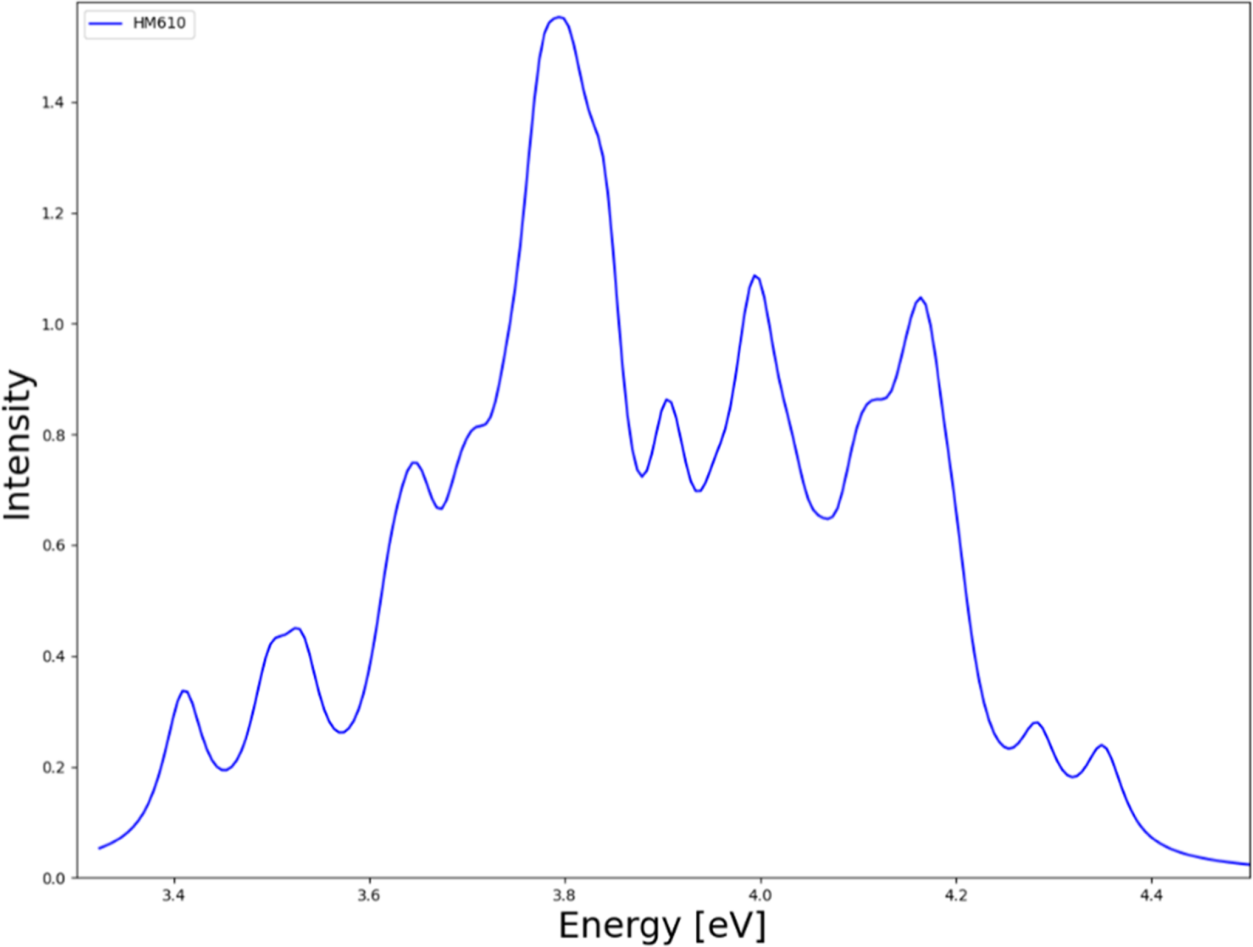
Simulated absorption spectrum of **HM610** computed at the BHLYP/def2-TZVP level of theory.

### Spin–Orbit Coupling Calculations for **HM610**

Spin**–**orbit couplings (SOCs) between the first eight singlets (including ground state S_0_) and ten triplets are computed by the relativistic two-component time-dependent sf-X2C–S-TD-DFT method employing M062X; ωB97; ωB97X; ωB97X-D; BHLYP; and CAM-B3LYP functionals. The magnitudes of spin–orbit couplings (SOCs) for all six functionals are presented in Tables 3–8, while the corresponding data for the M062X and CAM-B3LYP functionals in DMSO solvent are provided in Tables S9 and S10. There are only a few numbers of coupled states (states whose magnitude of SOCs is greater than or equal to 30 cm^–1^) in **HM610** that have been tabulated in Tables S11–S18 computed by all six functionals considered in this work. Appreciable magnitudes of SOCs in **HM610** will be explained by rules devised in our earlier work.^56^ According to them, when both the singlet state (φ_*i*_^*S*^→φ_*f*_^*S*^) and the triplet state (φ_*i*_^*T*^→φ_*f*_^*T*^) differ by a single pair of molecular orbitals or by both pairs, it is expected that these states of different multiplicity will exhibit strong spin–orbit coupling (SOC). First considering the case when singlet state (φ_*i*_^*S*^→φ_*f*_^*S*^) and triplet state (φ_*i*_^*T*^→φ_*f*_^*T*^) have either one of the orbitals different i.e.,

1 $$\varphi_{i}^{S}\neq \varphi_{i}^{T}$$

**Table 3:** Spin**–**Orbit Couplings (cm^–1^) between First Eight Singlets and Ten Triplets in **HM610** Employing M062X (TDDFT) Functional at the S_0_ Optimized Geometry

${Ĥ}_{DKH}$	|**T**_1_⟩	|**T**_2_⟩	|**T**_3_⟩	|**T**_4_⟩	|**T**_5_⟩	|**T**_6_⟩	|**T**_7_⟩	|**T**_8_⟩	|**T**_9_⟩	|**T**_10_⟩
⟨**S**_0_|	1	1	0	1	3	33	11	98	3	2
⟨**S**_1_|	0	0	0	1	1	7	2	15	1	0
⟨**S**_2_|	0	0	1	1	1	3	2	15	1	0
⟨**S**_3_|	14	13	36	9	7	0	9	8	3	8
⟨**S**_4_|	3	2	6	1	1	5	5	38	2	2
⟨**S**_5_|	10	2	7	3	2	1	1	2	12	6
⟨**S**_6_|	1	1	2	0	1	7	1	1	0	1
⟨**S**_7_|	1	1	1	0	1	7	0	1	0	1

**4:** Spin**–**Orbit Couplings (cm^–1^) between First Eight Singlets and Ten Triplets in **HM610** Employing ωB97 (TDDFT) Functional at the S_0_ Optimized Geometry

${Ĥ}_{DKH}$	|**T**_1_⟩	|**T**_2_⟩	|**T**_3_⟩	|**T**_4_⟩	|**T**_5_⟩	|**T**_6_⟩	|**T**_7_⟩	|**T**_8_⟩	|**T**_9_⟩	|**T**_10_⟩
⟨**S**_0_|	0	1	0	0	1	1	29	110	2	0
⟨**S**_1_|	1	0	1	1	0	1	7	14	1	0
⟨**S**_2_|	1	1	0	1	0	1	5	20	` 1	1
⟨**S**_3_|	0	0	1	0	0	1	6	37	1	1
⟨**S**_4_|	15	34	12	13	1	11	1	1	13	4
⟨**S**_5_|	8	14	6	11	2	7	0	1	10	1
⟨**S**_6_|	1	2	1	1	0	1	2	21	1	0
⟨**S**_7_|	0	1	0	0	0	1	6	17	1	0

**5:** Spin**–**Orbit Couplings (cm^–1^) between First Eight Singlets and Ten Triplets in **HM610** Employing ωB97X (TDDFT) Functional at the S_0_ Optimized Geometry

${Ĥ}_{DKH}$	|**T**_1_⟩	|**T**_2_⟩	|**T**_3_⟩	|**T**_4_⟩	|**T**_5_⟩	|**T**_6_⟩	|**T**_7_⟩	|**T**_8_⟩	|**T**_9_⟩	|**T**_10_⟩
⟨**S**_0_|	0	1	0	0	1	1	30	101	1	0
⟨**S**_1_|	1	0	1	1	0	1	7	15	1	0
⟨**S**_2_|	1	0	1	1	0	1	4	20	1	1
⟨**S**_3_|	0	1	1	0	0	1	7	38	1	1
⟨**S**_4_|	14	27	30	14	1	12	1	1	10	5
⟨**S**_5_|	11	3	4	7	2	3	0	1	11	1
⟨**S**_6_|	1	1	1	1	0	1	1	19	1	0
⟨**S**_7_|	1	1	1	0	0	1	7	11	1	0

**6:** Spin**–**Orbit Couplings (cm^–1^) between First Eight Singlets and Ten Triplets in **HM610** Employing ωB97X-D (TDDFT) Functional at the S_0_ Optimized Geometry

${Ĥ}_{DKH}$	|T_1_⟩	|T_2_⟩	|T_3_⟩	|T_4_⟩	|T_5_⟩	|T_6_⟩	|T_7_⟩	|T_8_⟩	|T_9_⟩	|T_10_⟩
⟨S_0_|	0	1	0	0	1	11	27	88	1	1
⟨S_1_|	0	0	1	1	0	3	6	16	1	0
⟨S_2_|	0	0	1	1	0	2	3	20	1	0
⟨S_3_|	2	2	4	1	1	3	6	39	1	1
⟨S_4_|	16	17	38	13	3	12	6	3	1	7
⟨S_5_|	1	1	2	1	0	2	3	17	1	1
⟨S_6_|	11	3	4	4	2	2	1	1	13	4
⟨S_7_|	1	1	1	0	0	4	7	4	1	1

**7:** Spin**–**Orbit Couplings (cm^–1^) between First Eight Singlets and Ten Triplets in **HM610** Employing CAM-B3LYP (TDDFT) Functional at the S_0_ Optimized Geometry

${Ĥ}_{DKH}$	|**T**_1_⟩	|**T**_2_⟩	|**T**_3_⟩	|**T**_4_⟩	|**T**_5_⟩	|**T**_6_⟩	|**T**_7_⟩	|**T**_8_⟩	|**T**_9_⟩	|**T**_10_⟩
⟨**S**_0_|	0	1	1	0	2	2	30	91	4	2
⟨**S**_1_|	1	0	1	1	0	1	7	16	1	0
⟨**S**_2_|	0	0	1	1	0	1	3	18	1	0
⟨**S**_3_|	3	4	6	2	1	2	7	40	3	2
⟨**S**_4_|	14	19	34	13	2	13	3	5	2	9
⟨**S**_5_|	1	2	4	1	0	2	4	16	1	1
⟨**S**_6_|	12	3	4	5	2	3	0	1	13	6
⟨**S**_7_|	1	1	1	0	0	1	8	4	0	1

**Table 8:** Spin**–**Orbit Couplings (cm^–1^) between First Eight Singlets and Ten Triplets in **HM610** Employing BHLYP (TDDFT) Functional at the S_0_ Optimized Geometry

${Ĥ}_{DKH}$	|T_1_⟩	|T_2_⟩	|T_3_⟩	|T_4_⟩	|T_5_⟩	|T_6_⟩	|T_7_⟩	|T_8_⟩	|T_9_⟩	|T_10_⟩
⟨S_0_|	0	1	0	0	1	1	31	96	9	2
⟨S_1_|	1	0	0	0	0	1	8	15	2	0
⟨S_2_|	1	1	0	1	0	1	3	14	2	0
⟨S_3_|	1	2	1	1	0	1	7	36	4	1
⟨S_4_|	10	35	2	11	1	11	2	3	5	11
⟨S_5_|	3	11	1	3	0	4	4	16	0	3
⟨S_6_|	13	3	3	7	2	3	0	1	11	8
⟨S_7_|	1	1	1	0	0	0	6	4	1	0

Or

2 $$\varphi_{f}^{S}\neq \varphi_{f}^{T}$$

Then, there will be appreciable SOC between these respective singlet and triplet states. Above-mentioned rules can be simply explained with Slater–Condon rules,^57^ i.e.,

3 $$|S\rangle =|...\varphi_{i}\varphi_{n}...\rangle$$

4 $$|T\rangle =|...\varphi_{j}\varphi_{n}...\rangle$$

5 $$\left\langle S\left|h_{SOC}\right|T\right\rangle =\left\langle \varphi_{i}\left|h_{SOC}\right|\varphi_{j}\right\rangle$$

If the singlet and triplet states differ by only one orbital, then the magnitude of the spin–orbit coupling (SOC) between them is given by the spin–orbit Hamiltonian *h*_SOC_, evaluated between the differing orbitals of the respective singlet and triplet states, as shown in eq 5. So, the first rule that says states of different multiplicities differ by a single pair of molecular orbitals will have appreciable SOC is well justified by the Slater–Condon rule. In the conventional Slater–Condon formulation, spin orbitals are used to construct Slater determinants. In the present discussion, however, we employ molecular orbitals (i.e., the spatial parts of spin orbitals) ″ ${\varphi_{z}|}_{z=i...n}$ ″ in eqs 3–5 as the spin dependence is explicitly treated through the spin–orbit Hamiltonian. Now, considering the case where the singlet state (φ_*i*_^*S*^→φ_*f*_^*S*^) and the triplet state (φ_*i*_^*T*^→φ_*f*_^*T*^) simultaneously differ by both pairs of orbitals, i.e.,^56^

6 $$\varphi_{i}^{S}\neq \varphi_{i}^{T}$$

and

7 $$\varphi_{f}^{S}\neq \varphi_{f}^{T}$$

in that case as well, there will be again significant spin–orbit coupling (SOC) between the singlet and triplet states. This rule can also be explained with Slater**–**Condon rules,^56^ i.e.,

8 $$|S\rangle =|...\varphi_{i}\varphi_{j}...\rangle$$

9 $$|T\rangle =|...\varphi_{k}\varphi_{l}...\rangle$$

$$\left\langle S\left|h_{SOC}\right|T\right\rangle =\left\langle \varphi_{i}\left|h_{SOC}\right|\varphi_{k}\right\rangle \left\langle \varphi_{j}|\varphi_{l}\right\rangle +\left\langle \varphi_{i}\left|h_{SOC}\right|\varphi_{l}\right\rangle \left\langle \varphi_{j}|\varphi_{k}\right\rangle +$$

10 $$\left\langle \varphi_{j}\left|h_{SOC}\right|\varphi_{k}\right\rangle \left\langle \varphi_{i}|\varphi_{l}\right\rangle +\left\langle \varphi_{j}\left|h_{SOC}\right|\varphi_{l}\right\rangle \left\langle \varphi_{i}|\varphi_{k}\right\rangle$$

11 $$\left\langle \varphi_{j}|\varphi_{l}\right\rangle =S_{jl}$$

12 $$\left\langle \varphi_{j}|\varphi_{k}\right\rangle =S_{jk}$$

13 $$\left\langle \varphi_{i}|\varphi_{l}\right\rangle =S_{il}$$

14 $$\left\langle \varphi_{i}|\varphi_{k}\right\rangle =S_{ik}$$

All these *Overlap matrix* in eq 10 would be zero if ${\varphi_{z}|}_{z=i...n}$ in eqs 11–14 were spin orbitals. Considering only one pair, let’s assume ⟨φ_*j*_|φ_*l*_⟩ = *S*_*jl*_ *overlap matrix* ″*S*_*jl*_^″^ will be nonzero i.e. *S*_*jl*_ ≠ 0 if ″φ_*j*_^″^ and ″φ_*l*_^″^ are drawn from two different (“state dependent”) orbital sets (one for bra determinant *S*, another for ket determinant *T*), thus resulting in a nonzero magnitude of spin–orbit coupling between respective singlet and triplet, i.e.,

15 $$\left\langle S\left|h_{SOC}\right|T\right\rangle \neq 0$$

in fact, we are working with a single set of orthonormal orbitals, so within that set *S*_*ij*_ = δ_*ij*_. The exact reasoning for nonzero SOC matrix elements between *S* and *T* states differing by more than one orbital is that these states are, in fact, CIS-like and could be expanded into multiple Slater determinants (though the expansion is, in most cases, dominated by the reference configuration).

16 $$|T>=\Sigma_{j}c_{T,j}|\Phi_{j}>$$

17 $$<S|=\Sigma_{i}c_{S,i}{\times <\Phi }_{i}|$$

Inserting such for both *S* and *T*, there could be a bilinear expression in

18 $$<S\left|h_{SOC}\right|T>=\Sigma_{ij}c_{T,j}c_{S,i}\times <\Phi_{i}\left|h_{SOC}\right|\Phi_{j}>$$

always with (several) terms where individual < Φ_*i*_|*h*_SOC_|Φ_*j*_ > are with Φ_*i*_ and Φ_*j*_ differing by a single orbital (consider monoexcitation from one of these “different orbitals in reference” into the same orbital). After that, there is only a single orbital difference, and normal Slater**–**Condon rules apply, resulting in <Φ_*i*_|*h*_SOC_|Φ_*j*_> ≠ 0 and thus (likely, except in the improbable case of mutual cancel-out) <*S*|*h*_SOC_|*T*> ≠0. We can introduce an “effective state-specific rotated” MO basis different for *S* and *T* (with some numerical error, not affecting the quality of the argument) and stick to the “nonorthogonal overlap” explanation, though. For convenience, coupled states differing by a single pair of molecular orbitals are referred to as “*true-coupled states*”, whereas those differing by both pairs of molecular orbitals are denoted as “*pseudo-coupled states*”. Now first consider coupled states computed by the M062X functional at the sf-X2C–S-TD-DFT-SOC/x2c-TZVPPall level of theory presented in Table S11. All coupled states computed by M062X functional can be explained by the first rule explained above, i.e., singlet and triplet state, differ by a single pair of molecular orbitals. So, they are all true-coupled states. Now considering coupled states computed by the ωB97 functional at the sf-X2C–S-TD-DFT-SOC/x2c-TZVPPall level of theory presented in Table S12. All coupled states computed by the ωB97 functional can be explained by the first rule mentioned above in eqs 1 and 2. Except for coupled state S_3_T_8_ (ωB97), this can be explained by the second rule explained above in light of Slater**–**Condon rules. Now considering S_3_T_8_ (ωB97) being pseudocoupled states whose respective singlet and triplet differ by both pairs of orbitals simultaneously, then one has to expand them into multiple Slater determinants, which will result in a bilinear expression differing only by a single orbital difference, and normal Slater**–**Condon rules apply. Now considering coupled states computed by ωB97X and ωB97X-D functionals at the sf-X2C–S-TD-DFT-SOC/x2c-TZVPPall level of theory presented in Tables S13 and S14. First, two coupled states are well explained by the first rule that if a singlet state (φ_*i*_^*S*^→φ_*f*_^*S*^) and triplet state (φ_*i*_^*T*^→φ_*f*_^*T*^) differ by just a single pair of orbitals, then they will result in true-coupled states. From Tables S13 and S14, it can be inferred that S_3_T_8_ and S_4_T_3_ coupled states of ωB97X and ωB97X-D functionals are pseudocoupled, which can be confirmed by expanding these states into multiple Slater determinants. Now considering coupled states computed by CAM-B3LYP and BHLYP functionals at the sf-X2C–S-TD-DFT-SOC/x2c-TZVPPall level of theory presented in Tables S15 and S16. The first three coupled states of the CAM-B3LYP functional being true-coupled states can be simply explained by the first rule.^52^ For pseudocoupled state S_4_T_3_ (CAM-B3LYP), one has to follow the procedure mentioned in eqs 16–18. Similarly, the first two and the fourth coupled states of the BHLYP functional can again be simply explained by the first rule.^56^ For the pseudocoupled state S_3_T_8_ (BHLYP), again, the same methodology mentioned above in eqs 16–18 has to be considered. Finally, considering coupled states computed by M062X and CAM-B3LYP functionals at sf-X2C–S-TD-DFT-SOC/x2c-TZVPP at all levels of theory in DMSO solvent employing the PCM solvation model presented in Tables S17 and S18. All coupled states can be explained in light of the first rule that singlet and triplet states differ by a single orbital pair.^56^ For the pseudocoupled state S_4_T_3_ (M062X and CAM-B3LYP), again bilinear expansion has to be considered. Thus, all coupled states computed by all six functionals (even in DMSO) summarized in Tables S11–S18 are well explained by rules devised earlier,^56^ which can be traced back to Slater–Condon rules, as shown in this work. The significant magnitude of spin–orbit coupling (SOC) observed when only a single pair of spin orbitals differs between singlet and triplet states has been previously reported.^6b,58^ However, a comprehensive justification for the occurrence of similarly large SOC values when both orbital pairs differ in singlet and triplet configurations is presented here for the first time, where CIS-like states should be expanded into multiple Slater determinants.^59^ When the orbitals involved in transitions between states of different multiplicities differ by their type (e.g., *n* → π* vs π → π*), the resulting large spin–orbit coupling (SOC) arises from an orientational change^60^ during the transition. In contrast, when the orbitals differ only by subscripts or energy levels (e.g., π_1_ → π* vs π_2_ → π*), the large SOC primarily results from a spatial change in the involved orbitals.^56a^ El-Sayed rules,^61^ normally employed to explain SOCs in organic molecules, is just a special case of the first rule explained above. Shapes of other molecular orbitals of **HM610** are also presented in Table S19. Thus, to comprehensively explain SOCs in a molecule, a threshold value for SOC (30 cm^–1^ in the present work) should be defined. States of different spin multiplicities exhibiting SOC values equal to or greater than this threshold can then be designated as coupled states. For such coupled states, the electronic character and dominant orbitals involved in the corresponding transitions should be explicitly analyzed.

### DMRG Results

DMRG calculations were performed to confirm that the system does not exhibit multireference character and that the chosen methodology is appropriate. Two indicators were used for this purpose: the occupation numbers of the DMRG natural orbitals and the single-orbital entropies. The latter quantify the importance of individual orbitals in the wave function expansion and have been previously employed in CAS-based analyses. The DMRG natural orbital occupations of 1.94 for the HOMO and 0.06 for the LUMO indicate a predominantly single-reference character, which is consistent with the single-orbital entropies reported in the Supporting Information in Table S20.

### Simulation of Dynamical Effects on Spin–Orbit Couplings in **HM610**

To explore the dynamical effects (first-order spin–vibronic effects) on SOCs trajectory surface hopping (TSH), simulations of **HM610** were run at the BHLYP-D3/dhf-TZVP level of theory. An ensemble of FSSH molecular dynamics (MD) simulations, consisting of 11 trajectories, was performed. The initial conditions, nuclear coordinates, and momenta were sampled from the Wigner distribution corresponding to a canonical ensemble of noninteracting quantum harmonic oscillators at the optimized ground-state geometry at 298 K. Each trajectory was propagated for 100 fs, employing a scaling factor of 5. A scaling approach for TSH simulations was employed since SOCs in **HM610** are not large enough with a few numbers of coupled states. Results of TSH simulations presented in Figure 2 clearly show that first-order spin–vibronic effects, i.e., dependence of SOCs on nuclear coordinates,^22a,22d^ cannot be an effective mechanism for inducing appreciable intersystem crossing in **HM610**. Even when a large scaling factor of 5 was used for SOCs, no appreciable filling of the triplet manifold was observed during the entire simulation run. To elucidate the contribution of individual singlet states to the increasing total singlet population observed in Figure 2, the average populations of each singlet state and the aggregated triplet population across all trajectories are presented in Figure 3. Figure 3 shows that within the singlet manifold, S_2_ is the primary contributor, corresponding to the second-brightest state. Apart from direct decay from S_1_ via fluorescence, there is a possibility that the molecule transfers part of its population to S_2_ through a radiationless transition.^62^ Despite this population transfer, the final decay pathway of S_2_ remains radiative relaxation to the ground state, typically occurring within 1–10 ns,^63^ consistent with known fluorescence time scales. Mixed quantum-classical dynamics methods, such as surface hopping, are computationally prohibitive for simulating these longer time scales associated with fluorescence. Therefore, the chosen TSH simulation time scale is appropriate and confirms the absence of significant triplet population transfer via spin–vibronic coupling.

**Figure 2:**
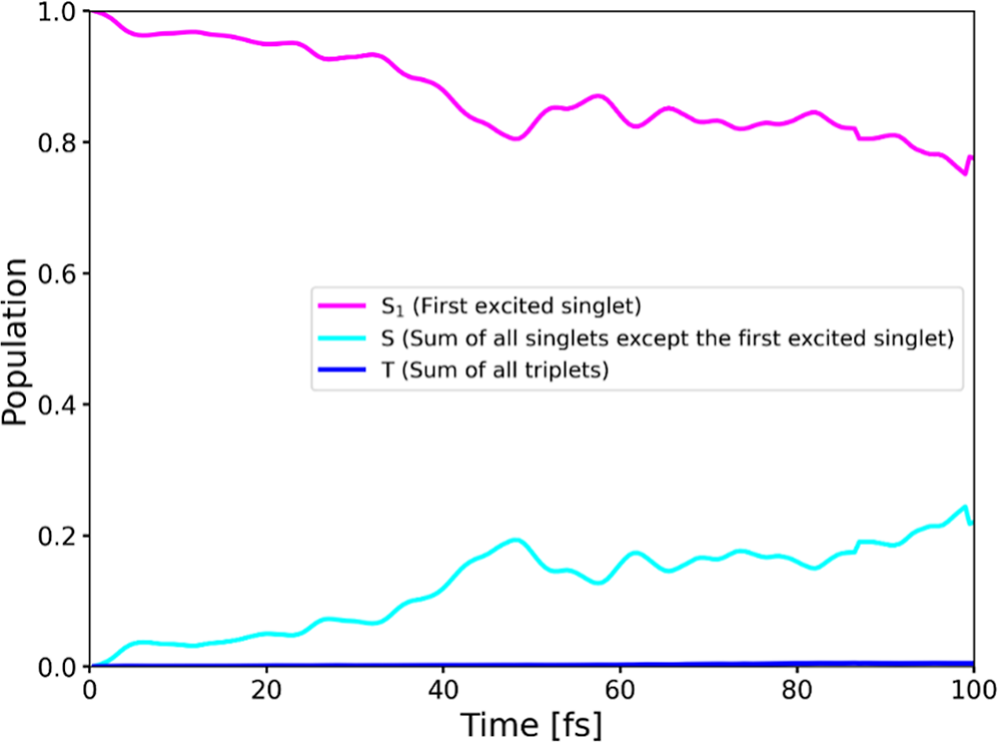
Average populations of singlets and triplets (back-transformed from the spin-adiabatic basis) in the dynamics of **HM610** started in the S_1_ state, with a scaling factor α = 5.

**Figure 3:**
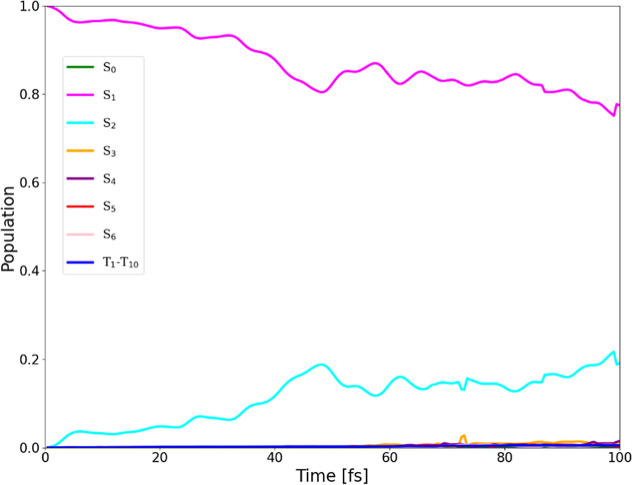
Average populations of individual singlet states and the combined triplet population (T_1_–T_10_) in the dynamics of **HM610**, which started in the S_1_ state, with a scaling factor α = 5.

### Calculation of NMR Chemical Shifts

The computed ^1^H and ^13^C NMR chemical shifts in both the gas phase and in CDCl_3_ (using PCM) are summarized in Tables S21 and S22. Overall, the experimental spectra exhibit close agreement with the theory, validating the structural assignments. For the ^1^H NMR spectra, the aromatic resonances (H21–H26) are well reproduced by all methods. The N–H proton (H22, 10.26 ppm) remains more deshielded in the experiment with deviations up to ∼1.8 ppm, though this error is slightly reduced with larger basis sets. Quantitatively, the RMSD analysis shows that B3LYP/Def2QZVPP (CDCl_3_) achieves the best agreement with the experiment (0.51 ppm), outperforming both the B3LYP/cc-pVQZ and M062X methods. The azomethine proton (H25, 8.27 ppm) is predicted within 0.5 ppm of experiment across all levels. Aliphatic protons (H29–H34) in the 3.2–4.2 ppm region are also consistently matched, confirming reliable treatment of methoxy and methylene environments. For the ^13^C NMR spectra, deshielded carbons such as the carbonyl C2 (170.7 ppm) and imine carbons C11 and C14 (∼153 ppm) are reproduced with reasonable accuracy, while aromatic carbons (C5, C8–C13, C17) are captured in the correct 110–140 ppm range. Methoxy carbons (C27, C28) are predicted near 56 ppm, with excellent consistency across all functionals. The RMSD analysis indicates that B3LYP/cc-pVQZ (CDCl_3_) provides the best overall agreement (8.43 ppm). These findings align with previous benchmarking, which established B3LYP as a reliable choice for NMR prediction in organic systems. Literature reports also suggest that M062X, particularly with larger basis sets such as Def2QZVPP, can offer improved accuracy for conjugated and hydrogen-bonded systems.^64^ However, in the present case, the M062X methods show significantly larger deviations (RMSD ≥ 18 ppm for ^13^C), indicating that they do not outperform B3LYP for this molecule. Importantly, our results demonstrate that basis set expansion within the B3LYP framework (Def2QZVPP, CDCl_3_) improves proton predictions, while B3LYP/cc-pVQZ (CDCl_3_) remains superior for carbons. The strong correlation between theory and experiment therefore validates the computational approach and provides robust support for the proposed molecular structure.

### Computational Exploration of **HM610** Analogues

To assess how donor–acceptor substitution modulates the geometry and photophysics of the benzylidenehydrazinylthiazole backbone, we constructed a systematic set of six derivatives (Figure 4). The series spans the unsubstituted parent (system 1), electron-donating OMe substitution at the aryl ring (systems 2 and 4), electron-withdrawing CF_3_ substitution at the thiazole ring (system 3), fully substituted analogues (systems 4 and 5), and experimentally synthesized compound **HM610** (system 6). Geometry optimizations were performed for both singlet and triplet states at the M062X/def2TZVP level, followed by TDDFT calculations of vertical excitations. All optimized geometries are provided in SI. Total SCF energies, zero-point vibrational energies, and thermal contributions to Gibbs energy are provided in Table S23. Results from TDDFT calculations (absorption wavelengths, energy gaps, and oscillator strengths) are condensed in Table S24.

**Figure 4:**
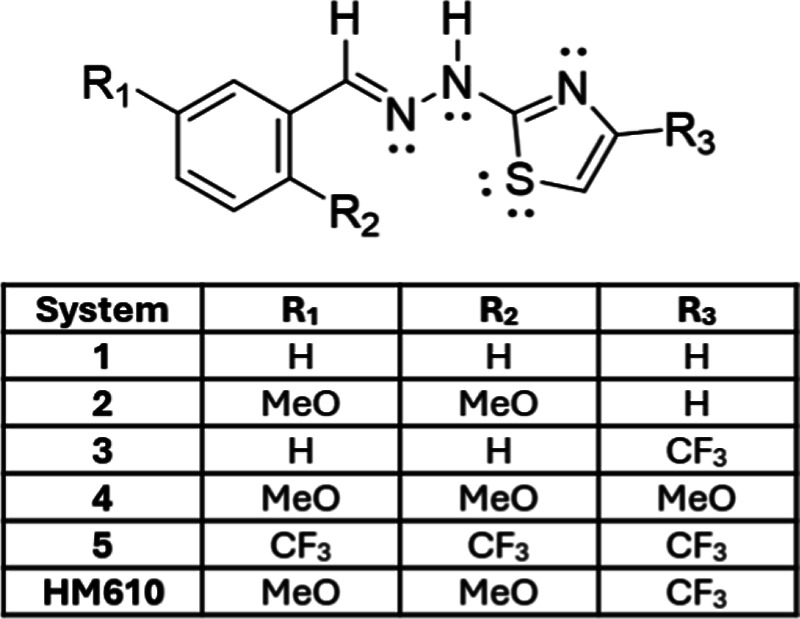
General structure of the benzylidenehydrazinylthiazole scaffold with variable substituents (R1–R3). The systematic set of six derivatives spans the unsubstituted parent (1), donor-substituted analogues (2, 4), acceptor-substituted analogues (3, 5), and the experimentally synthesized compound **HM610**.

The results show that substitution strongly influences singlet–triplet energetics and absorption profiles. OMe groups, as electron donors, generally red-shift the lowest–energy transitions and increase the oscillator strengths. In contrast, CF_3_ groups act as electron-withdrawing substituents, leading to larger blue-shifted excitations. The fully substituted analogues (systems 4 and 5) illustrate the extreme cases of donor- or acceptor-dominated substitution, while **HM610** (system 6) lies in between, combining donor and acceptor groups in a push–pull arrangement. Natural transition orbital (NTO) analysis illustrates how donor–acceptor substitution patterns modulate the photophysics of the studied thiazole–hydrazone derivatives. For the lowest singlet excitations (Figure 5), the excitation energies span 3.86 and 4.19 eV across the series. Systems bearing electron-donating OMe groups (2, 4, and **HM610**) display a modest red-shift relative to the unsubstituted parent (1), while strong electron-withdrawing substitution with CF_3_ (system 3) induces a slight blue-shift. The fully substituted analogues represent limiting cases: all-donor substitution (system 4) lowers the excitation energy to 3.86 eV, whereas all-acceptor substitution (system 5) maintains values close to those of the parent. Importantly, all molecules are essentially planar, allowing for maximal π overlap.

**Figure 5:**
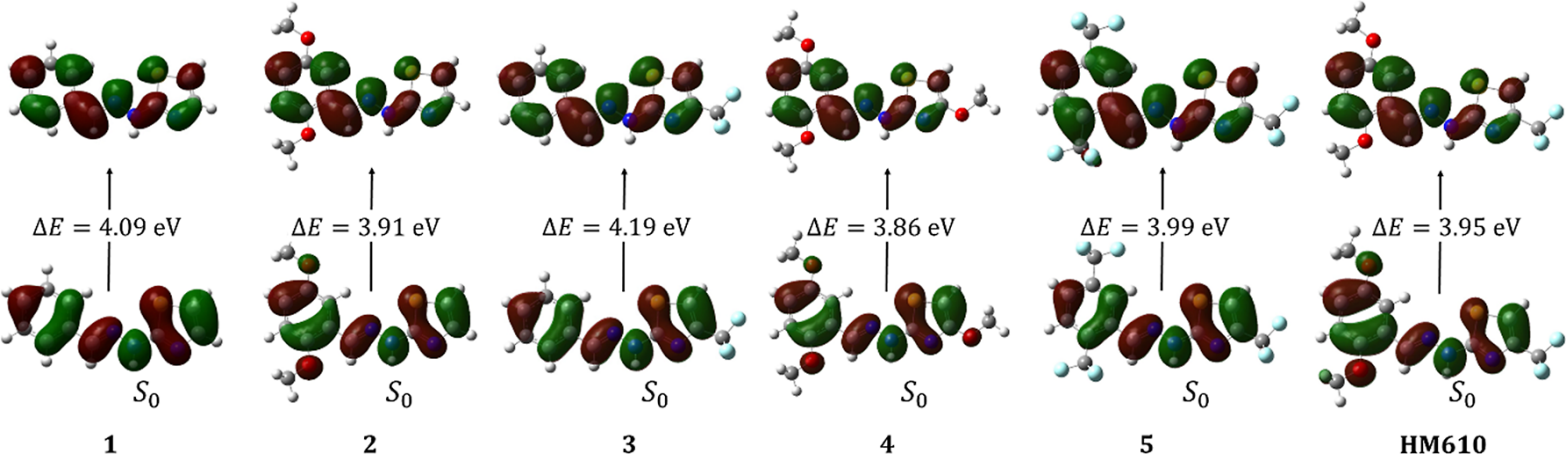
Natural transition orbitals representing the hole (bottom) and particle (top) orbitals for systems 1–5 and **HM610** in the singlet spin state, with associated excitation energies (Δ*E*, eV). All values were calculated at the td-M062X/def2TZVP level in vacuo, with 20 states in the CIS procedure.

The NTOs reveal that the lowest excitations are dominated by π→ π* transitions in every case, delocalized across the arylhydrazone-thiazole backbone, with substitution patterns affecting the relative localization of the hole and particle orbitals only slightly. The triplet state for all compounds is nonplanar excitations (Figure 6), and the relevant orbitals lie either on the thiazole subunit (compound 1) or on the phenyl ring (compounds 2–5 and **HM610**). For the lowest triplet, the series spans 2.67 and 3.45 eV. As in the singlet manifold, donor substitution tends to stabilize the triplet states, lowering excitation energies (systems 2, 4, and **HM610**). Conversely, CF_3_ substitution (system 3) increases energy gaps relative to the parent. In triplet space, the lowest energy gap is predicted for **HM610** (2.67 eV).

**Figure 6:**
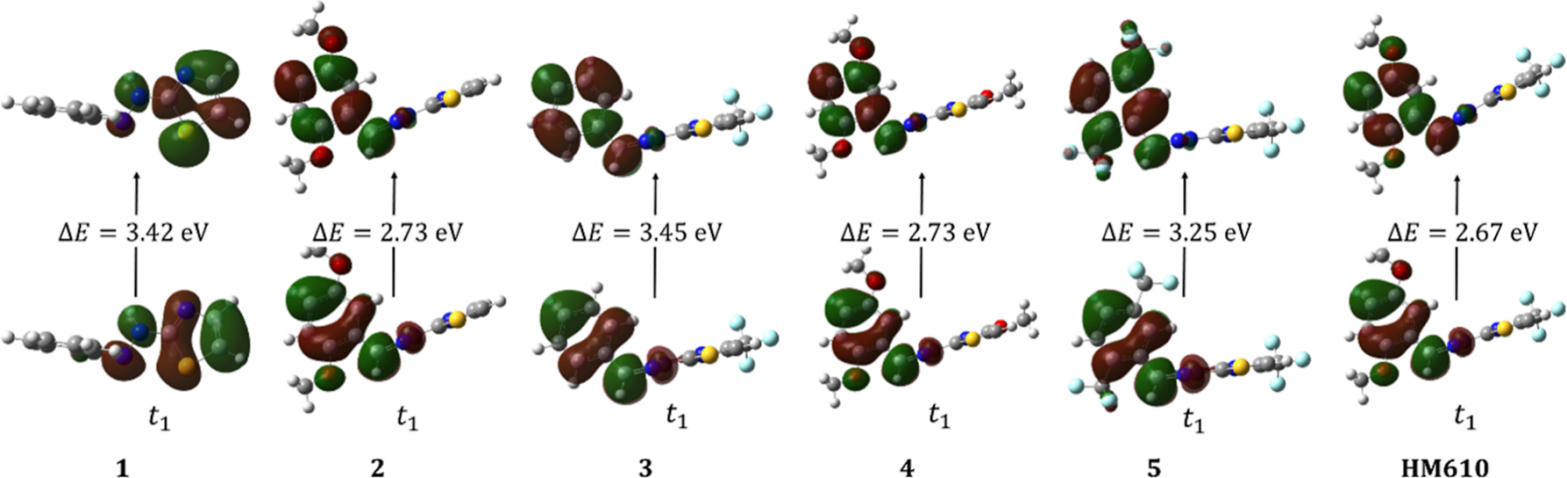
Natural transition orbitals representing the hole (bottom) and particle (top) orbitals for systems 1–5 and **HM610** in the triplet spin state, with associated excitation energies (Δ*E*, eV).

All values were calculated at the td-M062X/def2TZVP level in vacuo with 20 states in the CIS procedure. Overall, the systematic comparison of electron-donating and electron-withdrawing substitutions on the thiazole–hydrazone backbone shows that simple donor/acceptor modifications induce only moderate changes in excitation energies, oscillator strengths, and singlet–triplet gaps. Relativistic calculations confirm that spin–orbit couplings remain small, consistent with expectations for light-atom systems. These results indicate that, while substitution can fine-tune the photophysics of this scaffold, heavy-atom incorporation is likely essential to achieve efficient intersystem crossing and practical triplet sensitization. This perspective sets the stage for ongoing efforts aimed at introducing heavy substituents to enhance ISC in this family of chromophores.

## Conclusions

In this work, we established the synthetic feasibility of the thiazole–hydrazone scaffold through the preparation of **HM610** and applied a combined computational strategy to investigate its photophysics alongside a systematic set of analogues. Benchmarking TDDFT against correlated wave function methods (CC2 and ADC(2)) demonstrated close agreement in vertical excitation energies and state character, with M062X performing reliably relative to ωB97. Spin–orbit couplings (SOCs), evaluated with the sf-X2C–S-TDDFT method, were analyzed in terms of orbital contributions and found to follow the Slater–Condon rules: appreciable couplings occur when singlet and triplet states differ by either one pair or both pairs of orbitals. Consistently, the SOC magnitudes predicted for **HM610** are modest, suggesting a limited intersystem crossing efficiency in the absence of heavy-atom effects.

Our systematic DFT study of donor- and acceptor-substituted derivatives further revealed that electron-donating (−OMe) and electron-withdrawing (−CF_3_) groups produce only moderate shifts in excitation energies, oscillator strengths, and singlet–triplet gaps. Taken together, these findings indicate that heavy-atom substitution will likely be required to promote efficient triplet sensitization in this family of chromophores, providing clear motivation for ongoing studies directed at enhancing intersystem crossing by targeted functionalization.

## Supplementary Material



## Notes

Molecular structure, simulated spectrum, UV spectrum, IR spectrum, ^1^H-NMR spectrum, ^19^F-NMR spectrum, ^13^C-NMR spectrum of HM610, mass spectrum, vertical excitation energies, difference of vertical excitation energies, character of vertical excitation energies, spin-orbit couplings, coupled states, frontier molecular orbitals, orbital entropies, ^1^H chemical shifts, ^13^C chemical shifts, total SCF energies, zero-point vibrational energies, thermal contributions to Gibbs energy, TDDFT results, and Cartesian coordinates for all optimized molecular structures (PDF)
