# Supplementary material for: Slater–Condon Rules and Spin–Orbit Couplings: 2‑(2-(2,5-Dimethoxybenzylidene)hydrazineyl)-4-(trifluoromethyl)thiazole a Test Case
Source: ACS Omega. 2025 Nov 19;10(47):57776–89. doi: 10.1021/acsomega.5c10359 (PMC12676368; doi:10.1021/acsomega.5c10359)
Supplement: Supplementary file 1 [file ao5c10359_si_001.pdf]

Slater–Condon Rules and Spin–Orbit Couplings: 2-(2-(2,5-dimethoxybenzylidene)hydrazineyl)-4-(trifluoromethyl)thiazole a test case

Hasnain Mehmood,[1] Tashfeen Akhtar,[1] Jesús Espinosa-Romero,[2] Mauricio Maldonado-Domínguez,*[2] Jakub Višňák[3,4] and Mirza Wasif Baig*[4]

[1] Department of Chemistry, Mirpur University of Science and Technology (MUST), 10250-Mirpur (AJK) Pakistan.

[2] Facultad de Química, Departamento de Química Orgánica, Universidad Nacional Autónoma de México, 04510 Ciudad de México, México.

[3] Faculty of Mathematics and Physics, Charles University, Ke Karlovu 3, 12116 Prague, Czech Republic

[4] J. Heyrovský Institute of Physical Chemistry of the Czech Academy of Sciences, Dolejškova 2155/3, 18223 Prague 8, Czech Republic.

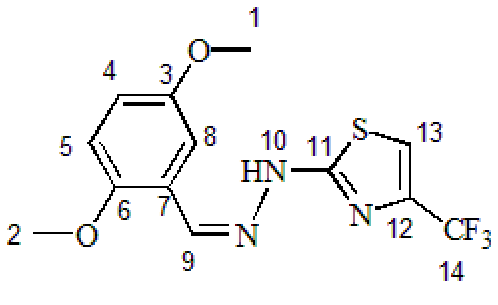

Figure S1: Molecular Structure of 2-(2-(2,5-dimethoxybenzylidene)hydrazineyl)-4-(trifluoromethyl)thiazole (HM610)

UV spectrum of HM610

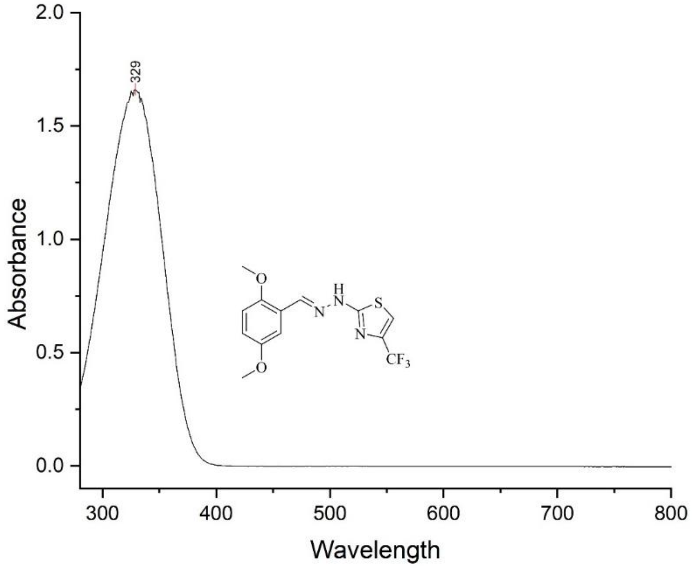

Simulated spectrum of HM610 in gas phase

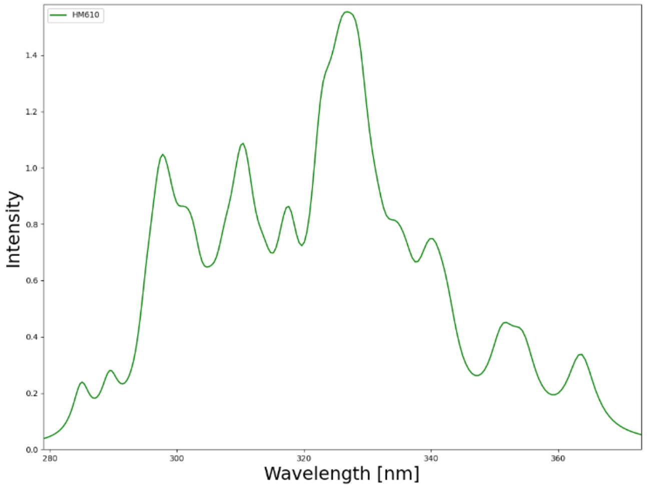

*** λ_{max} of HM610 for computed and simulated absorption almost matches i.e 329 nm**

IR spectrum of HM610

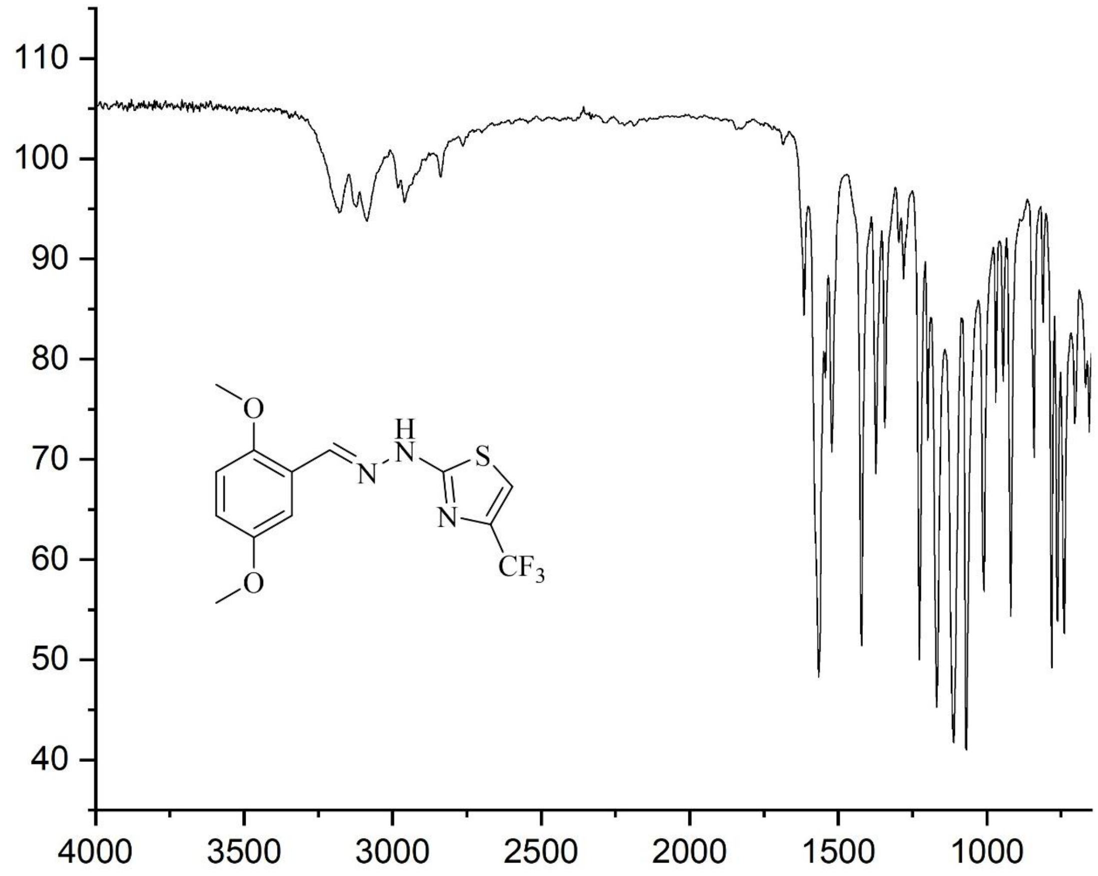

^{1}H-NMR spectrum of HM610

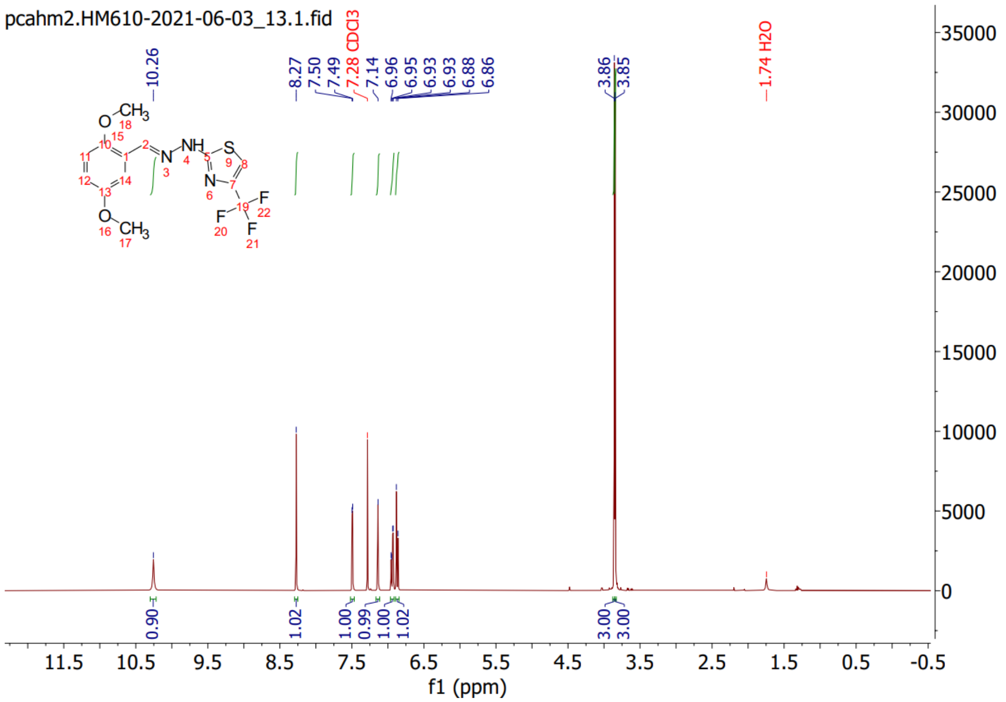

^{19}F-NMR spectrum of HM610

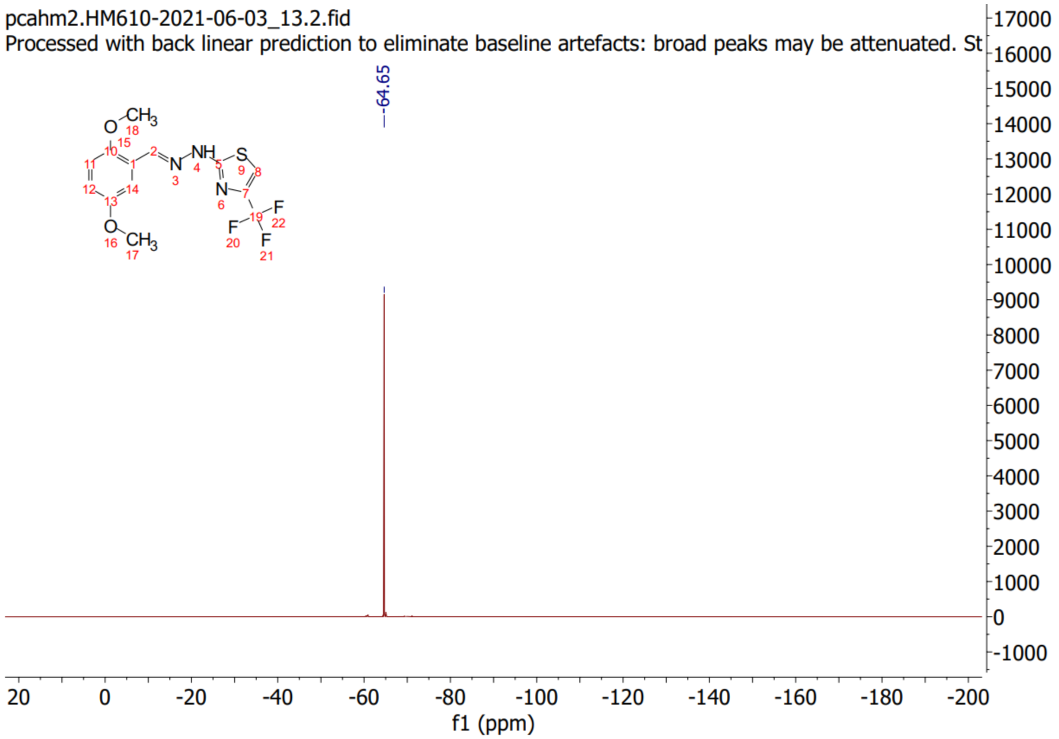

^{13}C-NMR spectrum of HM610

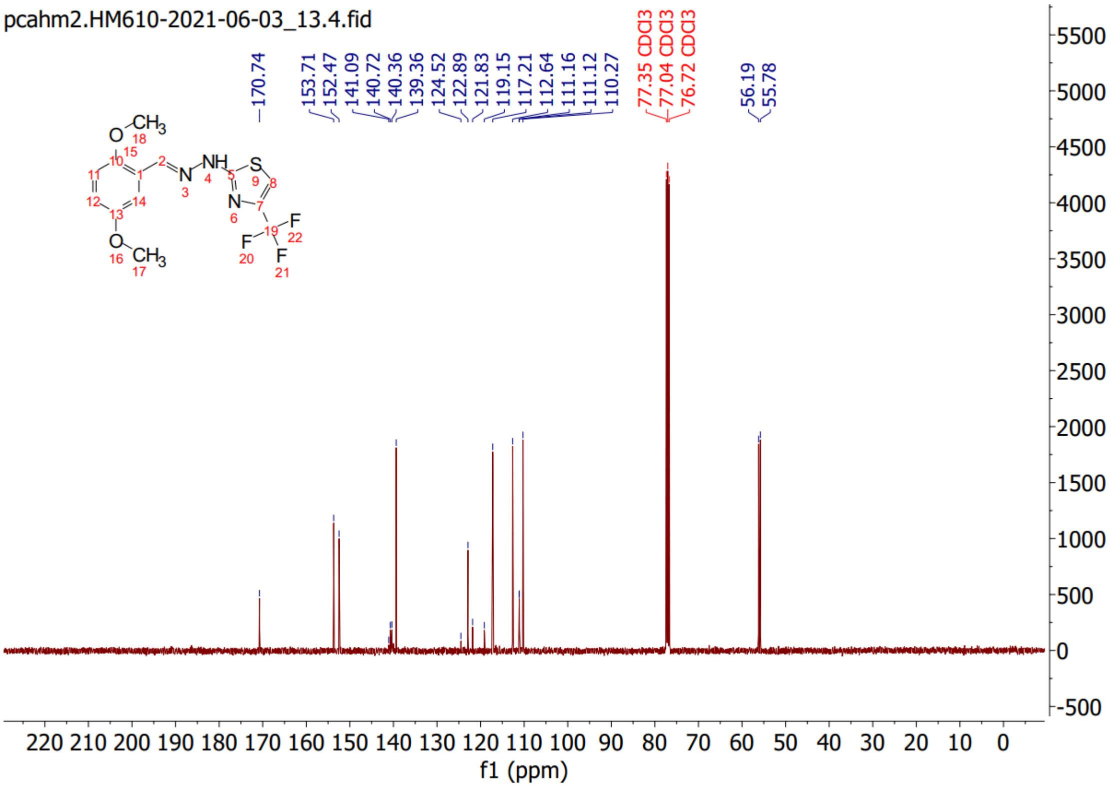

pcahm2.HM610-2021-06-03_13.4.fid
170.74
153.71
152.47
141.09
140.72
140.36
139.36
124.52
122.89
121.83
119.15
117.21
112.64
111.16
111.12
110.27
77.35 CDCl3
77.04 CDCl3
76.72 CDCl3
56.19
55.78
f1 (ppm)

Mass spectrum of HM610

Mass Spectrometry
Analytical Services
School of Chemistry

The University of Nottingham

Sample-ID	h_meh_HM610	Lab	C13
Submitter	Hasnain Mehmood (pcahm2)	Supervisor	Simon Woodward
Analysis Name	h_meh_HM610_616969_56_01_32724.d	Acquisition Date	6/9/2021 9:00:15 AM
Ionisation Mode	ESI Positive	Instrument	Bruker MicroTOF

+MS, 0.7-0.9min #41-52

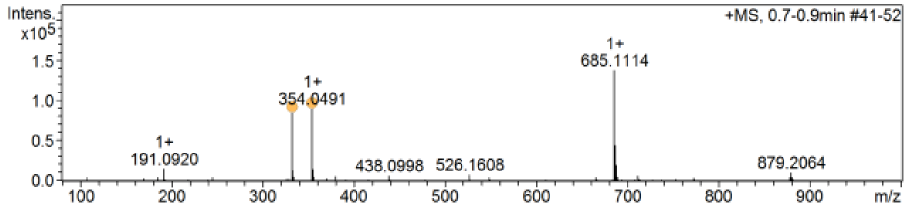

#	m/z	I %
1	107.0364	3.3
2	191.0920	11.1
3	245.1358	3.3
4	332.0674	61.5
5	333.0700	9.4
6	334.0654	3.6
7	354.0491	65.4
8	355.0522	10.7
9	356.0477	3.8
10	380.0859	4.0
11	438.0998	4.4
12	526.1608	5.7
13	685.1114	100.0
14	686.1142	32.6
15	687.1116	14.5
16	688.1119	3.5
17	711.1485	4.7
18	878.1110	3.2
19	879.2064	7.2
20	880.2092	3.2

Generate Molecular Formula Parameters

Charge	Tolerance	sigma limit	H/C Ratio	Electron Conf.	Nitrogen Rule	Chrom.BackGround	Calibration
+1	6 ppm	0.08	3 - 0	both	false	false	TRUE

Expected Formula C13 H12 F3 N3 O2 S1 **Adduct(s):** H, Na, NH4, C3H5N2, radical

#	meas. m/z	theo. m/z	|Err|[ppm]	Sigma	Formula	Adduct	Adduct Mass
1	332.0674	332.0675	0.40	0.0044	C13H13F3N3O2S	M+H	1.0078
1	354.0491	354.0495	0.90	0.0018	C13H12F3N3NaO2S	M+Na	22.9898

Note: Sigma fits < 0.05 indicates high probability of correct MF

Table S1: Vertical excitation energies of most important excited states computed with four different electronic structure methods at S_0 optimized geometry.

States	CC(2)	ADC(2)	M062X	ωB97
$(\pi;\pi^*)^1$	3.79 (0.50)	3.75 (0.46)	3.96 (0.66)	4.17 (0.61)
$(\pi;\sigma^*)^1$	5.44 (0.02)	5.47 (0.00)	5.43 (0.00)	5.85 (0.00)
$(\sigma;\pi^*)^1$	5.75 (0.00)	5.70 (0.00)	5.52 (0.00)	5.87 (0.00)
$(\pi;\pi^*)^3$	2.99 (0.00)	2.99 (0.00)	2.80 (0.00)	2.40 (0.00)
$(\pi;\sigma^*)^3$	5.28 (0.00)	5.32 (0.00)	5.25 (0.00)	5.51 (0.00)
$(\sigma;\pi^*)^3$	5.21 (0.00)	5.21 (0.00)	4.90 (0.00)	5.10 (0.00)

Table S2: Vertical excitation energies of most important excited states computed with four different electronic structure methods at S_0 optimized geometry.

States	CC(2)	ADC(2)	ωB97X	ωB97X-D
$(\pi;\pi^*)^1$	3.79 (0.50)	3.75 (0.46)	4.09 (0.62)	3.93 (0.61)
$(\pi;\sigma^*)^1$	5.44 (0.02)	5.47 (0.00)	5.70 (0.00)	5.48 (0.00)
$(\sigma;\pi^*)^1$	5.75 (0.00)	5.70 (0.00)	5.79 (0.00)	5.65 (0.12)
$(\pi;\pi^*)^3$	2.99 (0.00)	2.99 (0.00)	2.44 (0.00)	2.50 (0.00)
$(\pi;\sigma^*)^3$	5.28 (0.00)	5.32 (0.00)	5.40 (0.00)	5.22 (0.00)
$(\sigma;\pi^*)^3$	5.21 (0.00)	5.21 (0.00)	5.01 (0.00)	4.92 (0.00)

Table S3: Vertical excitation energies of most important excited states computed with four different electronic structure methods at S_0 optimized geometry.

States	CC(2)	ADC(2)	CAM-B3LYP	BHLYP
$(\pi;\pi^*)^1$	3.79 (0.50)	3.75 (0.46)	3.91 (0.61)	4.06 (0.68)
$(\pi;\sigma^*)^1$	5.44 (0.02)	5.47 (0.00)	5.45 (0.00)	5.60 (0.00)
$(\sigma;\pi^*)^1$	5.75 (0.00)	5.70 (0.00)	5.67 (0.00)	5.90 (0.00)
$(\pi;\pi^*)^3$	2.99 (0.00)	2.99 (0.00)	2.38 (0.00)	2.06 (0.00)
$(\pi;\sigma^*)^3$	5.28 (0.00)	5.32 (0.00)	5.17 (0.00)	5.27 (0.00)
$(\sigma;\pi^*)^3$	5.21 (0.00)	5.21 (0.00)	4.87 (0.00)	4.98 (0.00)

Table S4: Difference of vertical excitation energizes of HM610 between wave function methods (CC2 and ADC(2)) sf-XC-S-TD-DFT method (M062X and ωB97 functionals)

ADC(2)-CC2	M062X-CC2	ωB97-CC2	M062X-ADC(2)	ωB97-ADC(2)
-0.04	0.17	0.38	0.21	0.42
-0.05	0.19	0.41	0.24	0.46
-0.03	0.02	0.19	0.05	0.22
0.03	0.02	0.41	-0.01	0.38
-0.05	-0.06	0.29	-0.01	0.34
-0.05	-0.15	0.23	-0.1	0.28
-0.01	0.08	0.52	0.09	0.53
0	-0.19	-0.59	-0.19	-0.59
0	-0.09	-0.23	-0.09	-0.23
0	-0.14	-0.54	-0.14	-0.54
0	-0.11	-0.55	-0.11	-0.55
0	-0.1	-0.2	-0.1	-0.2
0	-0.31	-0.22	-0.31	-0.22
0	-0.15	-0.18	-0.15	-0.18
0	-0.07	0.19	-0.07	0.19
0	-0.25	-0.04	-0.25	-0.04
0	-0.26	-0.08	-0.26	-0.08

Table S5: Difference of vertical excitation energizes of HM610 between wave function methods (CC2 and ADC(2)) sf-XC-S-TD-DFT method (ωB97X and ωB97X-D functionals)

ADC(2)-CC2	ωB97X-CC2	ωB97X-D-CC2	ωB97X -ADC(2)	ωB97X-D-ADC(2)
-0.04	0.3	0.14	0.34	0.18
-0.05	0.33	0.16	0.38	0.21
-0.03	0.14	0.03	0.17	0.06
0.03	0.26	0.04	0.23	0.01
-0.05	0.21	0.07	0.26	0.12
-0.05	0.14	-0.09	0.19	-0.04
-0.01	0.38	0.11	0.39	0.12
0	-0.55	-0.49	-0.55	-0.49
0	-0.23	-0.28	-0.23	-0.28
0	-0.54	-0.49	-0.54	-0.49
0	-0.5	-0.43	-0.5	-0.43
0	-0.24	-0.29	-0.24	-0.29
0	-0.26	-0.3	-0.26	-0.3
0	-0.27	-0.36	-0.27	-0.36
0	0.08	-0.1	0.08	-0.1
0	-0.12	-0.26	-0.12	-0.26
0	-0.17	-0.31	-0.17	-0.31

Table S6: Difference of vertical excitation energizes of HM610 between wave function methods (CC2 and ADC(2)) sf-XC-S-TD-DFT method (CAM-B3LYP and BHLYP functionals)

ADC(2)-CC2	CAM-B3LYP-CC2	BHLYP-CC2	CAM-B3LYP -ADC(2)	BHLYP-ADC(2)
-0.04	0.12	0.27	0.16	0.31
-0.05	0.12	0.28	0.17	0.33
-0.03	0.01	0.09	0.04	0.12
0.03	0.01	0.16	-0.02	0.13
-0.05	-0.03	0.07	0.02	0.12
-0.05	-0.08	0.15	-0.03	0.2
-0.01	0.03	0.19	0.04	0.2
0	-0.61	-0.93	-0.61	-0.93
0	-0.33	-0.46	-0.33	-0.46
0	-0.6	-0.64	-0.6	-0.64
0	-0.56	-0.75	-0.56	-0.75
0	-0.35	-0.32	-0.35	-0.32
0	-0.38	-0.48	-0.38	-0.48
0	-0.41	-0.3	-0.41	-0.3
0	-0.15	-0.05	-0.15	-0.05
0	-0.36	-0.3	-0.36	-0.3
0	-0.4	-.0.27	-0.4	-0.27

Table S7: Difference of vertical excitation energizes of HM610 computed in gas phase and DMSO solvent using PCM model for M062X and CAM-B3LYP functionals

States	M062X	M062X (DMSO)	Difference	CAM-B3LYP	CAM-B3LYP (DMSO)	Difference
S_1	3.96 (0.66)	3.94 (0.62)	-0.02	3.91 (0.61)	3.88 (0.57)	-0.03
S_2	4.40 (0.09)	4.40 (0.14)	0.00	4.33 (0.14)	4.34 (0.18)	0.01
S_3	5.43 (0.00)	5.47 (0.11)	0.04	5.42 (0.07)	5.39 (0.09)	-0.03
S_4	5.46 (0.11)	5.52 (0.01)	0.06	5.45 (0.00)	5.49 (0.00)	0.04
S_5	5.52 (0.00)	5.55 (0.00)	0.03	5.55 (0.08)	5.54 (0.06)	-0.01
S_6	5.60 (0.00)	5.60 (0.03)	0.00	5.67 (0.00)	5.67 (0.00)	0.00
S_7	5.95 (0.04)	5.96 (0.20)	0.01	5.90 (0.20)	5.92 (0.17)	0.02
T_1	2.80 (0.00)	2.80 (0.00)	0.00	2.38 (0.00)	2.39 (0.00)	0.01
T_2	3.53 (0.00)	3.50 (0.00)	-0.03	3.29 (0.00)	3.26 (0.00)	-0.03
T_3	3.86 (0.00)	3.85 (0.00)	-0.01	3.40 (0.00)	3.39 (0.00)	-0.01
T_4	4.31 (0.00)	4.30 (0.00)	-0.01	3.86 (0.00)	3.87 (0.00)	0.01
T_5	4.71 (0.00)	4.72 (0.00)	0.01	4.46 (0.00)	4.46 (0.00)	0.00
T_6	4.90 (0.00)	4.92 (0.00)	0.02	4.83 (0.00)	4.76 (0.00)	-0.07
T_7	5.13 (0.00)	5.05 (0.00)	-0.08	4.87 (0.00)	4.90 (0.00)	0.03
T_8	5.25 (0.00)	5.29 (0.00)	0.04	5.17 (0.00)	5.21 (0.00)	0.04
T_9	5.47 (0.00)	5.52 (0.00)	0.05	5.36 (0.00)	5.41 (0.00)	0.05
T_{10}	5.63 (0.00)	5.68 (0.00)	0.05	5.49 (0.00)	5.54 (0.00)	0.05

Table S8: Character of vertical excitation energizes of HM610 computed in gas phase and DMSO solvent using PCM model for M062X and CAM-B3LYP functionals.

States	M062X	M062X (DMSO)	CAM-B3LYP	CAM-B3LYP (DMSO)
S_1	$(\pi;\pi^*)^1$	$(\pi;\pi^*)^1$	$(\pi;\pi^*)^1$	$(\pi;\pi^*)^1$
S_2	$(\pi;\pi^*)^1$	$(\pi;\pi^*)^1$	$(\pi;\pi^*)^1$	$(\pi;\pi^*)^1$
S_3	$(\pi;\sigma^*)^1$	$(\pi;\pi^*)^1$	$(\pi;\pi^*)^1$	$(\pi;\pi^*)^1$
S_4	$(\pi;\pi^*)^1$	$(\pi;\sigma^*)^1$	$(\pi;\sigma^*)^1$	$(\pi;\sigma^*)^1$
S_5	$(\sigma;\pi^*)^1$	$(\sigma;\pi^*)^1$	$(\pi;\pi^*)^1$	$(\pi;\pi^*)^1$
S_6	$(\pi;\pi^*)^1$	$(\pi;\pi^*)^1$	$(\sigma;\pi^*)^1$	$(\sigma;\pi^*)^1$
S_7	$(\pi;\sigma^*)^1$	$(\pi;\pi^*)^1$	$(\pi;\pi^*)^1$	$(\pi;\pi^*)^1$
T_1	$(\pi;\pi^*)^3$	$(\pi;\pi^*)^3$	$(\pi;\pi^*)^3$	$(\pi;\pi^*)^3$
T_2	$(\pi;\pi^*)^3$	$(\pi;\pi^*)^3$	$(\pi;\pi^*)^3$	$(\pi;\pi^*)^3$
T_3	$(\pi;\pi^*)^3$	$(\pi;\pi^*)^3$	$(\pi;\pi^*)^3$	$(\pi;\pi^*)^3$
T_4	$(\pi;\pi^*)^3$	$(\pi;\pi^*)^3$	$(\pi;\pi^*)^3$	$(\pi;\pi^*)^3$
T_5	$(\pi;\pi^*)^3$	$(\pi;\pi^*)^3$	$(\pi;\pi^*)^3$	$(\pi;\pi^*)^3$
T_6	$(\sigma;\pi^*)^3$	$(\sigma;\pi^*)^3$	$(\pi;\pi^*)^3$	$(\pi;\pi^*)^3$
T_7	$(\pi;\pi^*)^3$	$(\pi;\pi^*)^3$	$(\sigma;\pi^*)^3$	$(\sigma;\pi^*)^3$
T_8	$(\pi;\sigma^*)^3$	$(\pi;\sigma^*)^3$	$(\pi;\sigma^*)^3$	$(\pi;\sigma^*)^3$
T_9	$(\pi;\pi^*)^3$	$(\pi;\pi^*)^3$	$(\pi;\pi^*)^3$	$(\pi;\pi^*)^3$
T_{10}	$(\pi;\pi^*)^3$	$(\pi;\pi^*)^3$	$(\pi;\pi^*)^3$	$(\pi;\pi^*)^3$

Table S9: Spin-orbit couplings (cm^{-1}) in **HM610** employing M062X (TDDFT) functional at S_0 optimized geometry with PCM model (DMSO)

$\hat{H}_{DKH}$	$\|T_1\rangle$	$\|T_2\rangle$	$\|T_3\rangle$	$\|T_4\rangle$	$\|T_5\rangle$	$\|T_6\rangle$	$\|T_7\rangle$	$\|T_8\rangle$	$\|T_9\rangle$	$\|T_{10}\rangle$
$\langle S_0\|$	1	1	0	1	3	31	5	102	2	1
$\langle S_1\|$	0	0	0	1	1	7	1	15	1	0
$\langle S_2\|$	0	0	9	1	1	3	1	18	1	0
$\langle S_3\|$	3	3	7	2	1	4	2	37	2	1
$\langle S_4\|$	15	14	37	8	5	1	11	7	3	8
$\langle S_5\|$	9	2	7	2	2	0	2	1	12	6
$\langle S_6\|$	1	1	2	0	1	7	0	3	1	1
$\langle S_7\|$	1	1	1	0	1	7	0	3	0	0

Table S10: Spin-orbit couplings (cm^{-1}) in **HM610** employing CAM-B3LYP (TDDFT) functional at S_0 optimized geometry with PCM model (DMSO)

$\hat{H}_{DKH}$	$\|T_1\rangle$	$\|T_2\rangle$	$\|T_3\rangle$	$\|T_4\rangle$	$\|T_5\rangle$	$\|T_6\rangle$	$\|T_7\rangle$	$\|T_8\rangle$	$\|T_9\rangle$	$\|T_{10}\rangle$
$\langle S_0\|$	0	1	1	0	2	1	28	95	2	1
$\langle S_1\|$	1	0	1	1	0	1	7	16	1	0
$\langle S_2\|$	0	0	1	1	0	1	3	21	1	0
$\langle S_3\|$	1	1	1	1	0	1	6	41	2	1
$\langle S_4\|$	16	19	37	13	0	16	1	0	3	8
$\langle S_5\|$	1	2	5	2	0	2	5	8	0	1
$\langle S_6\|$	12	4	4	5	1	3	0	1	13	7
$\langle S_7\|$	1	1	0	0	0	1	8	6	0	0

Table S11: Coupled states in HM610 computed at M062X/x2c-TZVPPall level of theory on top of S_0 optimized geometry

Coupled states	Orbitals	Character	|SOC|
S_0T_6	$(85; 79 \rightarrow 86)$	$(\pi)^1;(\sigma;\pi^*)^3$	33
S_0T_8	$(85; 85 \rightarrow 89)$	$(\pi)^1; (\pi;\sigma^*)^3$	98
S_3T_3	$(85 \rightarrow 89; 85 \rightarrow 87)$	$(\pi;\sigma^*)^1; (\pi;\pi^*)^3$	36
S_4T_8	$(85 \rightarrow 87; 85 \rightarrow 89)$	$(\pi;\pi^*)^1; (\pi;\sigma^*)^3$	38

Table S12: Coupled states in HM610 computed at *ω*B97/x2c-TZVPPall level of theory on top of S_0 optimized geometry

Coupled states	Orbitals	Character	|SOC|
S_0T_7	$(85; 79 \rightarrow 86)$	$(\pi)^1;(\sigma;\pi^*)^3$	29
S_0T_8	$(85; 85 \rightarrow 89)$	$(\pi)^1; (\pi;\sigma^*)^3$	110
S_3T_8	$(83 \rightarrow 86; 85 \rightarrow 89)$	$(\pi;\pi^*)^1; (\pi;\sigma^*)^3$	37
S_4T_2	$(85 \rightarrow 89; 85 \rightarrow 87)$	$(\pi;\sigma^*)^1;(\pi;\pi^*)^3$	34

Table S13: Coupled states in HM610 computed at ωB97X/x2c-TZVPPall level of theory on top of S_0 optimized geometry

Coupled states	Orbitals	Character	|SOC|
S_0T_7	$(85; 79 \rightarrow 86)$	$(\pi)^1;(\sigma;\pi^*)^3$	30
S_0T_8	$(85; 85 \rightarrow 89)$	$(\pi)^1; (\pi;\sigma^*)^3$	101
S_3T_8	$(83 \rightarrow 86; 85 \rightarrow 89)$	$(\pi;\pi^*)^1; (\pi;\sigma^*)^3$	38
S_4T_3	$(85 \rightarrow 89; 84 \rightarrow 87)$	$(\pi;\sigma^*)^1;(\pi;\pi^*)^3$	30

Table S14: Coupled states in HM610 computed at ωB97X-D/x2c-TZVPPall level of theory on top of S_0 optimized geometry

Coupled states	Orbitals	Character	|SOC|
S_0T_7	$(85; 79 \rightarrow 86)$	$(\pi)^1;(\sigma;\pi^*)^3$	27
S_0T_8	$(85; 85 \rightarrow 89)$	$(\pi)^1; (\pi;\sigma^*)^3$	88
S_3T_8	$(83 \rightarrow 86; 85 \rightarrow 89)$	$(\pi;\pi^*)^1; (\pi;\sigma^*)^3$	39
S_4T_3	$(85 \rightarrow 89; 84 \rightarrow 87)$	$(\pi;\sigma^*)^1;(\pi;\pi^*)^3$	38

Table S15: Coupled states in HM610 computed at CAM-B3LYP/x2c-TZVPPall level of theory on top of S_0 optimized geometry

Coupled states	Orbitals	Character	|SOC|
S_0T_7	$(85; 79 \rightarrow 86)$	$(\pi)^1;(\sigma;\pi^*)^3$	30
S_0T_8	$(85; 85 \rightarrow 89)$	$(\pi)^1; (\pi;\sigma^*)^3$	91
S_3T_8	$(85 \rightarrow 87; 85 \rightarrow 89)$	$(\pi;\pi^*)^1; (\pi;\sigma^*)^3$	40
S_4T_3	$(85 \rightarrow 89; 84 \rightarrow 87)$	$(\pi;\sigma^*)^1;(\pi;\pi^*)^3$	34

Table S16: Coupled states in HM610 computed at BHLYP/x2c-TZVPPall level of theory on top of S_0 optimized geometry

Coupled states	Orbitals	Character	|SOC|
S_0T_7	$(85; 79 \rightarrow 86)$	$(\pi)^1;(\sigma;\pi^*)^3$	31
S_0T_8	$(85; 85 \rightarrow 89)$	$(\pi)^1; (\pi;\sigma^*)^3$	96
S_3T_8	$(83 \rightarrow 86; 85 \rightarrow 89)$	$(\pi;\pi^*)^1; (\pi;\sigma^*)^3$	36
S_4T_2	$(85 \rightarrow 89; 85 \rightarrow 87)$	$(\pi;\sigma^*)^1;(\pi;\pi^*)^3$	34

Table S17: Coupled states in HM610 computed at M062X/x2c-TZVPPall level of theory on top of S_0 optimized geometry employing PCM model (DMSO)

Coupled states	Orbitals	Character	|SOC|
S_0T_6	$(85; 79 \rightarrow 86)$	$(\pi)^1;(\sigma;\pi^*)^3$	31
S_0T_8	$(85; 85 \rightarrow 89)$	$(\pi)^1; (\pi;\sigma^*)^3$	102
S_3T_3	$(85 \rightarrow 87; 84 \rightarrow 87)$	$(\pi;\pi^*)^1; (\pi;\pi^*)^3$	37
S_4T_3	$(85 \rightarrow 89; 84 \rightarrow 87)$	$(\pi;\sigma^*)^1; (\pi;\pi^*)^3$	38

Table S18: Coupled states in HM610 computed at CAM-B3LYP/x2c-TZVPPall level of theory on top of S_0 optimized geometry employing PCM model (DMO)

Coupled states	Orbitals	Character	|SOC|
S_0T_7	$(85; 79 \rightarrow 86)$	$(\pi)^1;(\sigma;\pi^*)^3$	28
S_0T_8	$(85; 85 \rightarrow 89)$	$(\pi)^1; (\pi;\sigma^*)^3$	95
S_3T_8	$(85 \rightarrow 87; 85 \rightarrow 89)$	$(\pi;\pi^*)^1; (\pi;\sigma^*)^3$	41
S_4T_3	$(85 \rightarrow 89; 84 \rightarrow 87)$	$(\pi;\sigma^*)^1;(\pi;\pi^*)^3$	37

Table S19: Frontier Molecular Orbitals of HM610 ranging from HOMO-7 to LUMO+3 computed with M062X and ωB97 functionals.

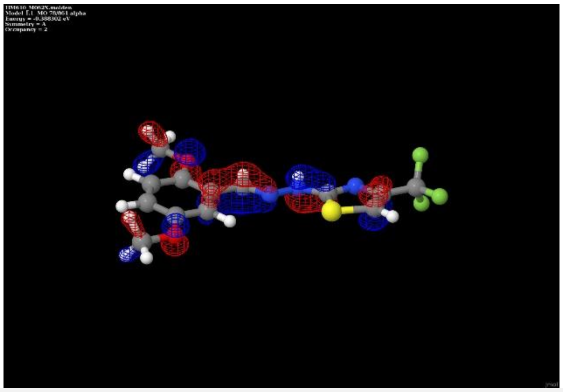

HOMO-7 (M062X) (orbital:78)

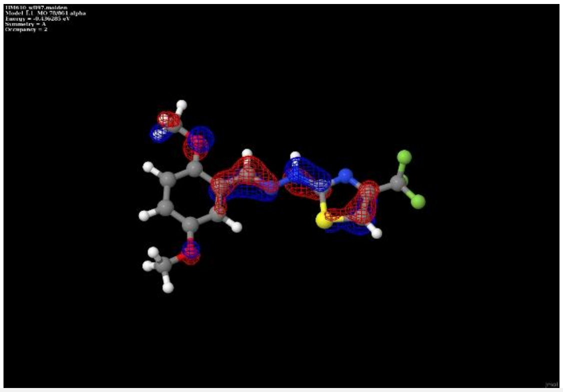

HOMO-7 (ωB97) (orbital:78)

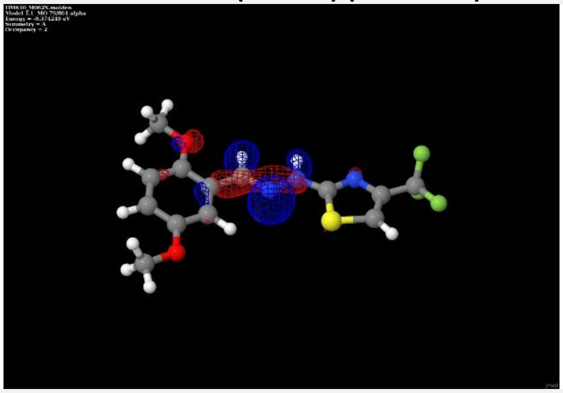

HOMO-6 (M062X) (orbital:79)

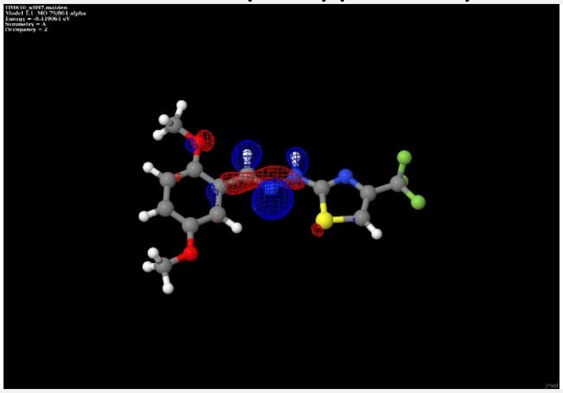

HOMO-6 (ωB97) (orbital:79)

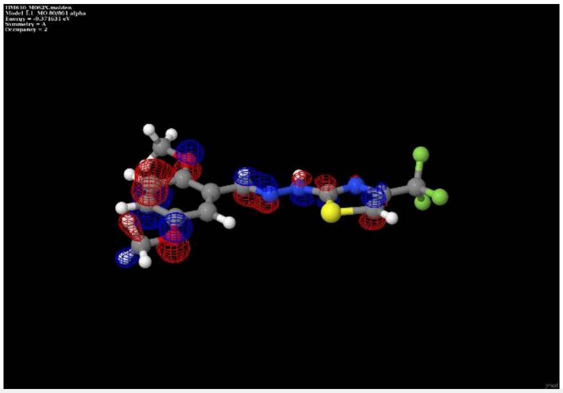

HOMO-5 (M062X) (orbital:80)

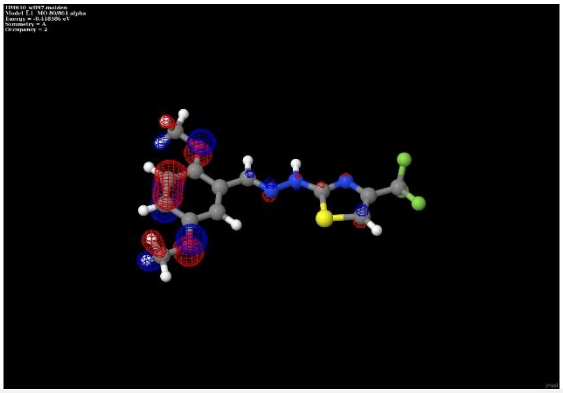

HOMO-5 (ωB97) (orbital:80)

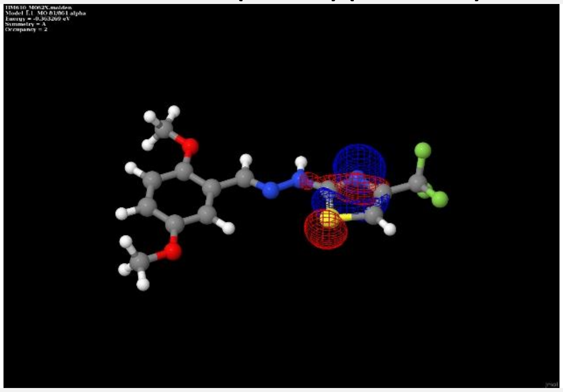

HOMO-4 (M062X) (orbital:81)

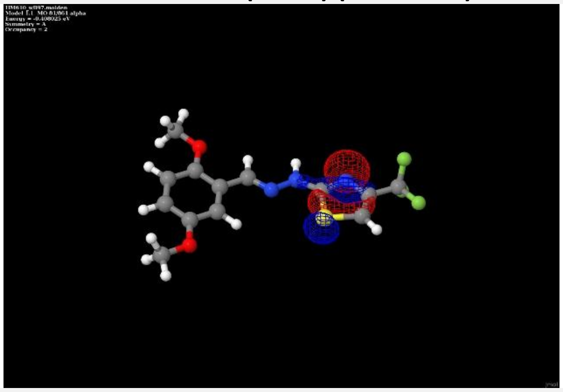

HOMO-4 (ωB97) (orbital:81)

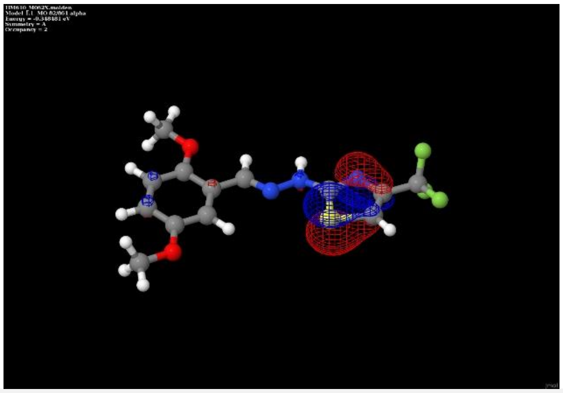

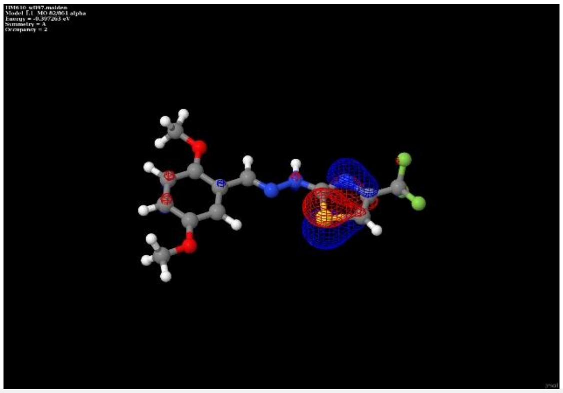

HOMO-3 (M062X) (orbital:82) HOMO-3 (ωB97) (orbital:82)

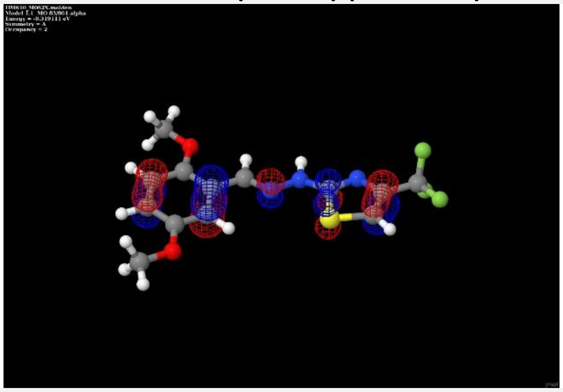

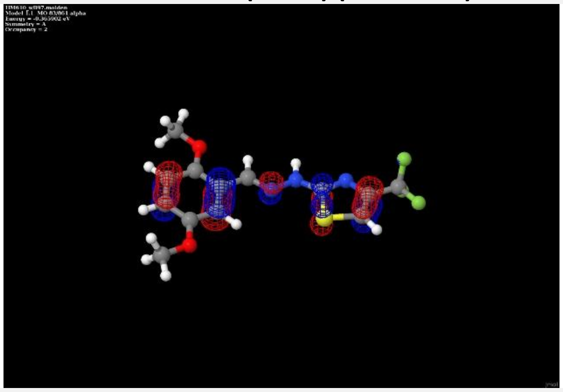

HOMO-2 (M062X) (orbital:83) HOMO-2 (ωB97) (orbital:83)

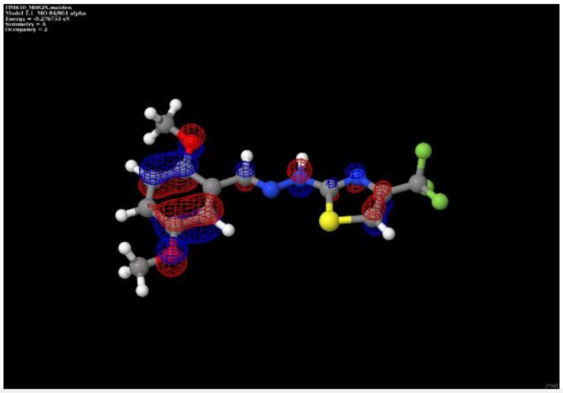

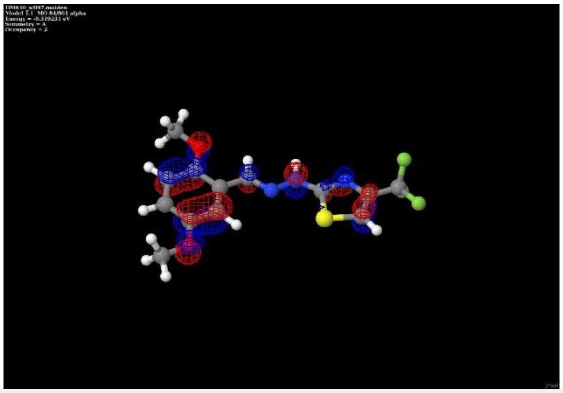

HOMO-1 (M062X) (orbital:84)

HOMO-1 (ωB97) (orbital:84)

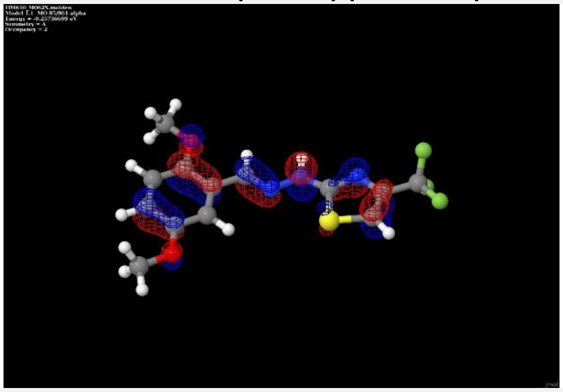

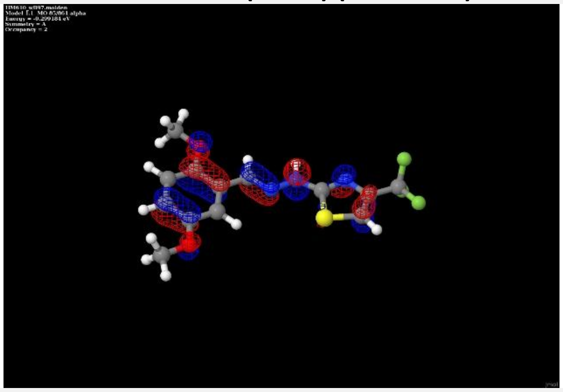

HOMO (M062X) (orbital:85)

HOMO (ωB97) (orbital:85)

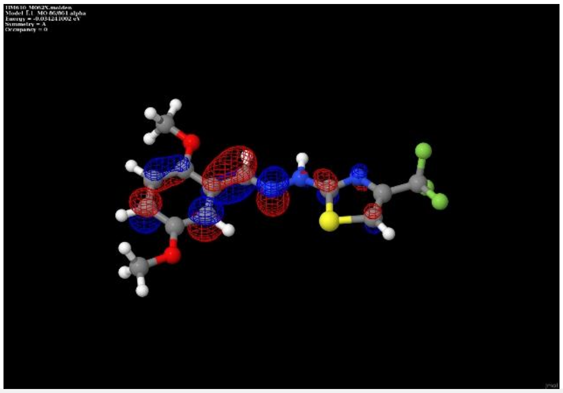

LUMO (M062X) (orbital:86)

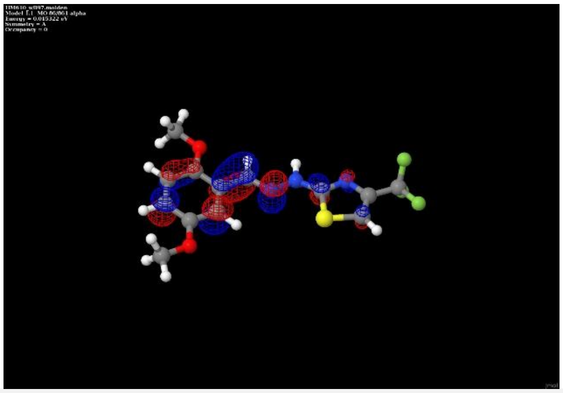

LUMO (ωB97) (orbital:86)

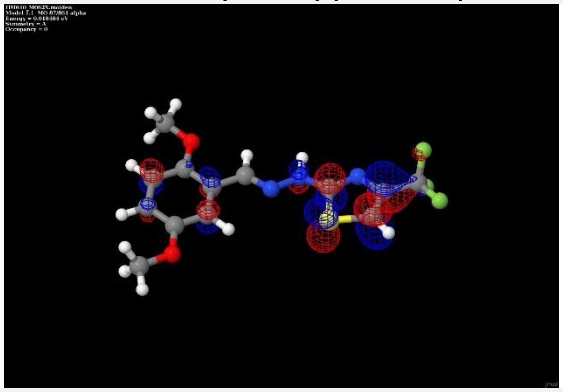

LUMO+1 (M062X) (orbital:87)

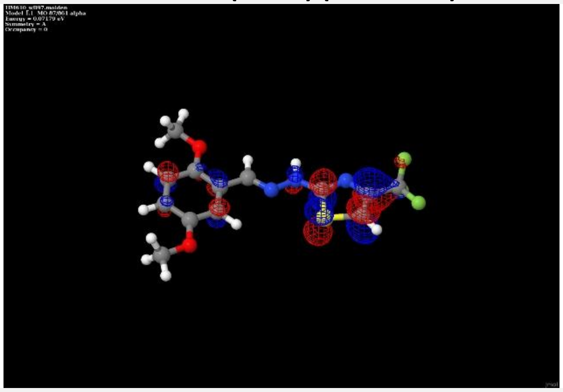

LUMO+1 (ωB97) (orbital:87)

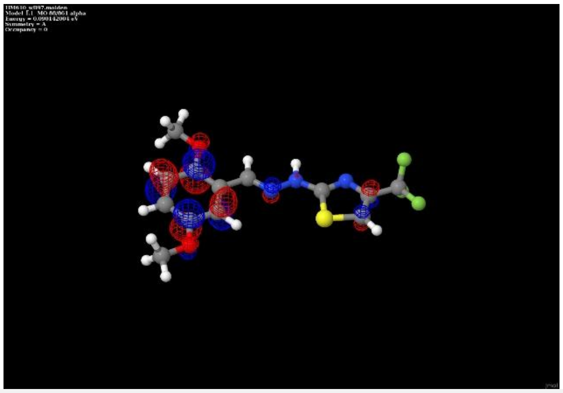

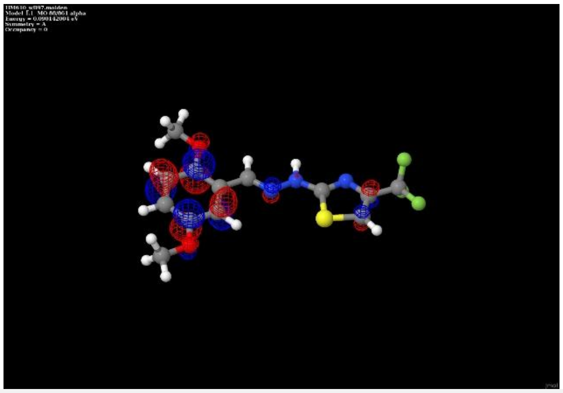

LUMO+2 (M062X) (orbital:88) LUMO+2 (ωB97) (orbital:88)

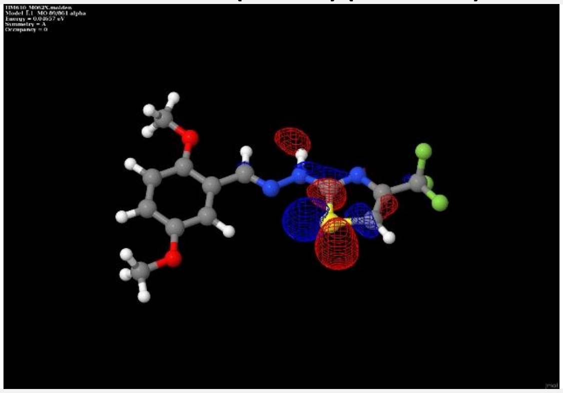

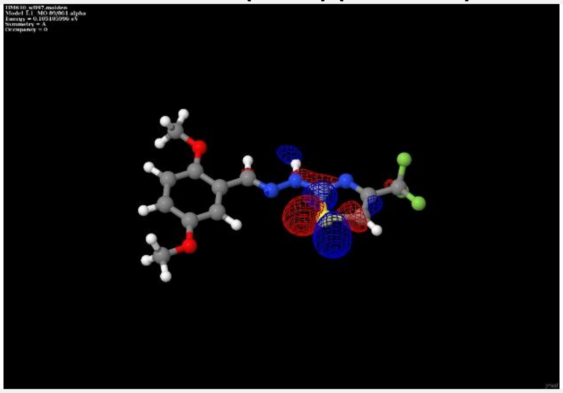

LUMO+3 (M062X) (orbital:89) LUMO+3 (ωB97) (orbital:89)

Table S20: Orbital entropies in HM610 computed by DMRG.

Orbital Entropies
0.00443976
0.00306474
0.08831756
0.01637977
0.08739358
0.03228039
0.11237942
0.21611789
0.18910291
0.21714082
0.23206128
0.19493116
0.18837806
0.02623684
0.00347528
0.11460917
0.03853718
0.01489764
0.11628968
0.00417322

Table S21: ^{1}H Chemical shifts computed at different DFT level of theories.

Number of atoms	Chemical Shift B3LYP/cc-pVQZ	Chemical Shift B3LYP/cc-pVQZ (CDCl3)	Chemical Shift B3LYP/ Def2QZVPP (CDCl3)	Chemical Shift M062X/cc-pVQZ (CDCl3)	Chemical Shift M062X/ Def2QZVPP (CDCl3)	Chemical shift (Exp)
H21	6.92	7.13	7.12	7.67	7.62	6.87
H22	8.41	8.69	8.73	9.12	9.14	10.26 $^{N-H}$
H23	6.94	7.14	7.15	7.69	7.62	6.94
H24	8.09	8.01	8.02	8.59	8.58	7.49
H25	8.39	8.51	8.54	8.92	8.93	8.27 $^{(Azomethine)}$
H26	7.11	7.29	7.29	7.76	7.74	7.14
H29	4.10	4.14	4.14	4.12	4.09	3.85
H30	3.68	3.77	3.77	3.71	3.68	3.85
H31	3.69	3.78	3.77	3.70	3.67	3.85
H32	4.15	4.22	4.23	4.20	4.20	3.86
H33	3.76	3.84	3.83	3.77	3.76	3.86
H34	3.72	3.78	3.78	3.71	3.70	3.86

***Numbering of atoms mentioned in above table is shown in Fig S2**

Table S22: ^{13}C Chemical shifts computed at different DFT level of theories.

Number of atoms	Chemical Shift B3LYP/cc-pVQZ	Chemical Shift B3LYP/cc-pVQZ (CDCl3)	Chemical Shift B3LYP/ Def2QZVPP (CDCl3)	Chemical Shift M062X/cc-pVQZ (CDCl3)	Chemical Shift M062X/ Def2QZVPP (CDCl3)	Chemical shift (Exp)
C2	179.33	179.66	180.29	180.82	178.5	170.7
C4	154.52	152.21	152.73	163.00	163.8	140.7
C5	118.83	120.08	120.03	133.58	134.2	112.2
C8	142.68	143.86	144.19	157.94	159.0	139.4
C9	133.81	132.64	132.80	145.09	145.3	119.2
C10	122.95	120.82	120.91	135.01	136.0	117.2
C11	165.40	164.30	164.78	175.73	177.2	152.5
C12	117.36	118.35	118.54	131.79	133.3	111.2
C13	115.95	116.97	117.16	129.79	130.0	110.3
C14	162.45	162.20	162.70	173.50	174.8	153.7
C17	133.52	133.32	133.89	130.14	131.0	122.4
C27	57.90	58.20	58.50	57.59	57.7	56.2
C28	57.89	58.18	58.45	57.46	57.2	55.8

***Numbering of atoms mentioned in above table is shown in Fig S2**

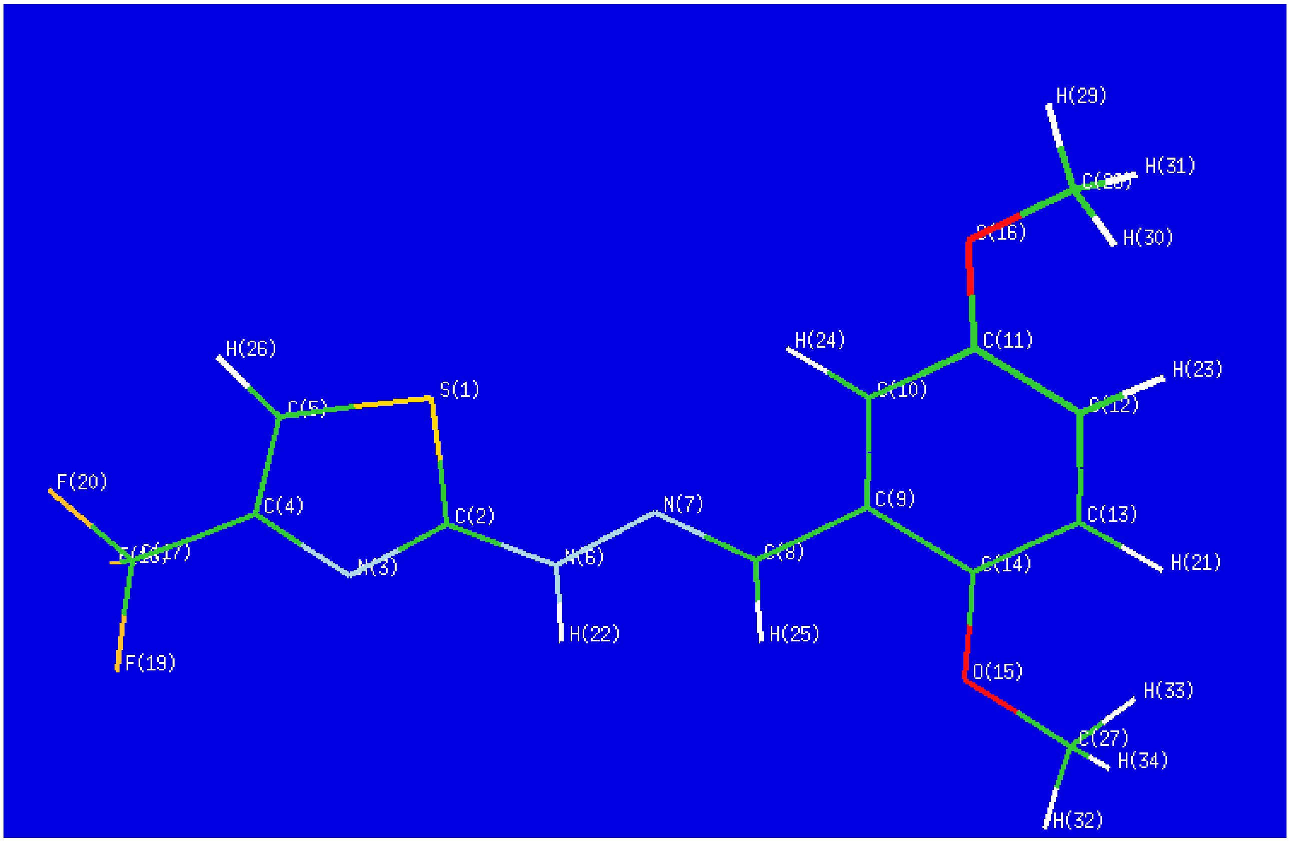

Figure S2: Molecular Structure of 2-(2-(2,5-dimethoxybenzylidene)hydrazineyl)-4-(trifluoromethyl)thiazole (HM610)

Table S23. Total SCF energies, zero-point vibrational energies, and thermal contributions to Gibbs energy for all compounds presented in Figure 4, in their optimized geometries for singlet and triplet spin states, calculated at the DFT M062X/def2-TZVP level.

	**R1**	**R2**	**R3**	**Spin multiplicity**	**Energy (UM062X) (Ha)**	**Zero-Point Energy (Ha)**	**Thermal corrections to Gibbs Free Energy (Ha)**
1	H	H	H	Singlet	-948.8627945	0.175892	0.135563
				Triplet	-948.7845603	0.173495	0.132338
2	MeO	MeO	H	Singlet	-1177.911932	0.242013	0.194759
				Triplet	-1177.835133	0.239616	0.191807
3	H	H	CF_3	Singlet	-1285.955572	0.180998	0.135033
				Triplet	-1285.876031	0.178636	0.131574
4	MeO	MeO	MeO	Singlet	-1292.439815	0.275162	0.224756
				Triplet	-1292.362617	0.272786	0.221683
5	CF_3	CF_3	CF_3	Singlet	-1960.135994	0.191412	0.133838
				Triplet	-1960.058465	0.189069	0.130746
HM610	MeO	MeO	CF_3	Singlet	-1515.005122	0.247077	0.194281
				Triplet	-1514.927018	0.244779	0.191186

Table S24. TDDFT results for all compounds presented in Figure 4, in their optimized geometries for singlet and triplet spin states, calculated at the TDDFT M062X/def2-TZVP level employing 20 states in the CIS procedure.

	**R1**	**R2**	**R3**	**Spin multiplicity**	**Excited state number**	**ΔE (eV)**	**λ (nm)**	**f**
1	H	H	H	Singlet	3	4.0935	302.88	0.6832
				Triplet	1	3.4186	362.68	0.0476
2	MeO	MeO	H	Singlet	4	3.9091	317.17	0.6881
				Triplet	1	2.7283	454.44	0.0433
3	H	H	CF_3	Singlet	3	4.187	296.12	0.7283
				Triplet	1	3.4502	359.35	0.0115
4	MeO	MeO	MeO	Singlet	4	3.8552	321.60	0.6612
				Triplet	1	2.7257	454.86	0.0468
5	CF_3	CF_3	CF_3	Singlet	3	3.9917	310.60	0.7080
				Triplet	1	3.2499	381.50	0.0066
HM610	MeO	MeO	CF_3	Singlet	4	3.9503	313.86	0.6645
				Triplet	1	2.6728	463.88	0.0444

Cartesian Coordinates for All Optimized Molecular Structures

HM610 S_0 optimized at TD-DFT/M062X/def2-TZVP

34

-1515.00504436076 a.u.

S	1.9374634	1.4365523	-0.0554943
C	1.9349104	-0.2841726	0.1666034
N	3.0975361	-0.8523622	0.2104556
C	4.0678925	0.1003335	0.0643642
C	3.6598396	1.3780652	-0.0881238
N	0.7697263	-0.9777184	0.2908500
N	-0.4075626	-0.3448321	0.1377513
C	-1.4652586	-1.0536384	0.1316404
C	-2.7858695	-0.4427872	-0.0202593
C	-2.9531518	0.9320466	-0.0063417
C	-4.2110665	1.5031716	-0.1625311
C	-5.3158121	0.6807794	-0.3342626
C	-5.1601212	-0.7038089	-0.3478148
C	-3.9083099	-1.2730335	-0.1909824
O	-3.6723901	-2.6137404	-0.1967318
O	-4.2595930	2.8607301	-0.1310650
C	5.4974538	-0.3435744	0.0729779
F	5.8241006	-0.9481021	1.2180206
F	5.7528357	-1.2071688	-0.9135105
F	6.3234122	0.6981029	-0.0819878
H	-6.0348834	-1.3219269	-0.4848226
H	0.8308184	-1.9884818	0.3417999
H	-6.3062306	1.0932585	-0.4583699
H	-2.0937122	1.5750693	0.1287120
H	-1.4203076	-2.1380741	0.2294925
H	4.2598705	2.2616713	-0.2184088
C	-4.7710694	-3.4722908	-0.3977424
C	-5.5182621	3.4709762	-0.2907173
H	-5.3466962	4.5425381	-0.2363544
H	-5.9591983	3.2235732	-1.2606701
H	-6.2076753	3.1738830	0.5048737
H	-4.3755904	-4.4840013	-0.3717852
H	-5.5163094	-3.3539179	0.3932024
H	-5.2408527	-3.2898019	-1.3679959

TMS S_0 optimized at TD-DFT/ M062X/def2-TZVP

17

-449.14541374160 a.u.

Si	0.1206952	-0.0000004	-0.0006788
C	0.2868928	0.0000025	1.8656390
H	1.3358349	0.0000224	2.1673030
H	-0.1858740	-0.8816975	2.3019654
H	-0.1859078	0.8816838	2.3019665
C	-1.6950432	0.0000017	-0.4638507
H	-1.8274672	0.0000233	-1.5472662
H	-2.2008358	-0.8818112	-0.0665127
H	-2.2008434	0.8817937	-0.0664761
C	0.9459081	1.5293841	-0.7013140
H	0.8891577	1.5413654	-1.7912267
H	0.4677422	2.4386202	-0.3325636
H	2.0001955	1.5698412	-0.4209524
C	0.9459040	-1.5293879	-0.7013106
H	0.8891528	-1.5413719	-1.7912232
H	2.0001913	-1.5698482	-0.4209493
H	0.4677347	-2.4386211	-0.3325575

Compound 1 S_0 from Figure 4 optimized at DFT/M062X/def2-TZVP

23

-948.8627945 a.u.

C	-0.99523400	0.12566200	-0.52672000
C	-2.13890000	0.85404300	-0.20736300
C	-2.00697500	2.06655000	0.47374200
C	-0.75323800	2.53236900	0.82377700
C	0.38561500	1.79972900	0.50173500
C	0.26177200	0.59558300	-0.17410100
H	-2.89741700	2.62968800	0.72028600
H	1.36438300	2.16916000	0.77833900
H	1.14286600	0.02080300	-0.42764300
C	-3.44828300	0.32830500	-0.59454500
H	-3.46721600	-0.62784400	-1.12570600
N	-4.52578100	0.95106300	-0.32817500
N	-5.68990000	0.41244300	-0.71230700
C	-6.87094500	1.04688000	-0.44100100
S	-6.91038200	2.55974300	0.39758000
N	-8.01768100	0.55979600	-0.79116700
C	-8.63171800	2.52722600	0.26037200
C	-9.02110400	1.40770900	-0.38910500
H	-9.23883600	3.32124700	0.65950400
H	-5.73685800	-0.47308800	-1.20310100
H	-10.04560500	1.14435200	-0.60662400
H	-1.09262800	-0.81567600	-1.05519500
H	-0.65832300	3.47256600	1.35144700

Compound 1 T_1 from Figure 4 optimized at DFT/M062X/def2-TZVP

23

-948.7845603 a.u.

C	-0.96216900	0.26708600	-0.87223600
C	-2.20644600	0.90600600	-0.67608700
C	-2.31099400	1.85664500	0.36309700
C	-1.22348700	2.13933300	1.16497700
C	-0.00639100	1.49389600	0.96305900
C	0.11497600	0.55782400	-0.06250600
H	-3.25205500	2.36994500	0.51399600
H	0.84124900	1.72011100	1.59585100
H	1.06031600	0.05669300	-0.22636700
C	-3.30980800	0.58242100	-1.50259000
H	-3.18767800	-0.10266200	-2.33611000
N	-4.53827200	1.22623200	-1.39322400
N	-5.37345200	0.66310100	-0.53404500
C	-6.62126300	1.18973200	-0.31137800
S	-7.15148800	2.60752300	-1.13828900
N	-7.45905000	0.66143400	0.52497500
C	-8.63851000	2.47869800	-0.27834600
C	-8.61223200	1.40044200	0.54229200
H	-9.43202200	3.19119000	-0.42558900
H	-5.11858500	-0.16581500	-0.00351700
H	-9.42051800	1.09587100	1.19022200
H	-0.86558100	-0.45988600	-1.66982700
H	-1.31892700	2.87248700	1.95567100

Compound 2 S_0 from Figure 4 optimized at DFT/M062X/def2-TZVP

31

-1177.911932 a.u.

C	-0.90154300	0.10868300	-0.45209900
C	-2.06971700	0.84187700	-0.17351300
C	-1.96262600	2.07090900	0.45658400
C	-0.72367700	2.58867800	0.81683000
C	0.42606400	1.86242700	0.54068700
C	0.33163300	0.62464700	-0.09323800
H	-2.85632800	2.64051700	0.67399100
H	1.40336700	2.23669800	0.80765600
H	1.23989100	0.07753600	-0.29810100
C	-3.37098500	0.29071100	-0.55452900
H	-3.38374400	-0.67941600	-1.05061100
N	-4.44818400	0.92630500	-0.31194300
N	-5.60933100	0.37123500	-0.68461100
C	-6.79272200	1.01020700	-0.44122400
S	-6.83939500	2.55091400	0.34550400
N	-7.93749100	0.50901500	-0.78005200
C	-8.56017300	2.50986600	0.20150900
C	-8.94410700	1.36806700	-0.41150000
H	-9.17100800	3.31530400	0.57097500
H	-5.64944300	-0.53057300	-1.14541600
H	-9.96714100	1.09528400	-0.62456300
O	-0.73900800	3.80239600	1.42978800
C	0.49849000	4.35440700	1.80999600
H	0.27614800	5.31015800	2.27689500
H	1.01600900	3.71202500	2.52849900
H	1.14450600	4.51692500	0.94224200
O	-1.07456200	-1.09183000	-1.07318600
C	0.07611300	-1.84876600	-1.36657800
H	0.61965500	-2.11137900	-0.45483500
H	-0.27124200	-2.75602300	-1.85355400
H	0.74399000	-1.30712100	-2.04201000

Compound 2 T_1 from Figure 4 optimized at DFT/M062X/def2-TZVP

31

-1177.835133 a.u.

C	-0.88141900	0.04344000	-0.72944200
C	-2.11769000	0.75201200	-0.66564600
C	-2.16198700	1.94124300	0.07966100
C	-1.04202100	2.41853400	0.74339800
C	0.15401100	1.71384800	0.67557800
C	0.22286600	0.52704500	-0.06501200
H	-3.08053100	2.51084600	0.13037400
H	1.04105100	2.06269400	1.18252200
H	1.16389700	-0.00181200	-0.10403900
C	-3.25276700	0.25060300	-1.33626600
H	-3.18051200	-0.63111400	-1.96041900
N	-4.46343600	0.93931100	-1.34296700
N	-5.25404300	0.65018600	-0.32075000
C	-6.48038600	1.24954600	-0.18305700
S	-7.03728200	2.41276900	-1.32890100
N	-7.27823200	0.98347900	0.80377100
C	-8.47693400	2.55515200	-0.39332200
C	-8.41905800	1.73168100	0.68142200
H	-9.26813600	3.22456600	-0.68452800
H	-4.97853600	-0.01373100	0.39800200
H	-9.19445900	1.63094800	1.42616300
O	-1.20684900	3.58168100	1.43053200
C	-0.08914900	4.10150300	2.10927600
H	-0.42100900	5.02060400	2.58490500
H	0.26974400	3.40732400	2.87477600
H	0.72726800	4.32658700	1.41687700
O	-0.89865500	-1.09736700	-1.46507400
C	0.30015200	-1.82944200	-1.56504400
H	0.63713900	-2.17019500	-0.58213300
H	0.08124400	-2.69021500	-2.19107300
H	1.08915700	-1.23204200	-2.02999100

Compound 3 S_0 from Figure 4 optimized at DFT/M062X/def2-TZVP

	26		
	-1285.955572 a.u.		
C	-1.01613900	0.16549100	-0.63743100
C	-2.14582200	0.86564900	-0.22057800
C	-1.98792100	2.05231300	0.49895100
C	-0.72076400	2.52158700	0.79094200
C	0.40433600	1.81751300	0.37144800
C	0.25432400	0.63900400	-0.34309500
H	-2.86788800	2.59320800	0.82118000
H	1.39373700	2.18968400	0.60277700
H	1.12509800	0.08723600	-0.67184900
C	-3.46922100	0.33579300	-0.54940300
H	-3.50700900	-0.60158900	-1.11189200
N	-4.53723400	0.93240800	-0.20045000
N	-5.71500700	0.38285800	-0.53867900
C	-6.87991400	0.99425600	-0.18444500
S	-6.87869800	2.48565200	0.70137300
N	-8.04380600	0.51139100	-0.48072400
C	-8.60111800	2.44958600	0.66557400
C	-9.01254100	1.34546400	0.00662600
H	-9.19945600	3.22075300	1.11877800
H	-5.77633300	-0.48554700	-1.05797400
H	-0.60443100	3.44158800	1.34885900
H	-1.13469800	-0.75584500	-1.19586700
C	-10.44476500	0.97411100	-0.22368400
F	-10.74566000	-0.20437900	0.32741600
F	-10.73061900	0.88411100	-1.52483800
F	-11.26664700	1.88751300	0.30662700

Compound 3 T_1 from Figure 4 optimized at DFT/M062X/def2-TZVP

26

-1285.876031 a.u.

C	-1.00518700	0.47952100	-1.01427200
C	-2.25943800	1.06348800	-0.73001000
C	-2.42160700	1.72786200	0.50564500
C	-1.37891800	1.79118800	1.40787000
C	-0.15148100	1.20277000	1.11494100
C	0.02665100	0.54883300	-0.10294400
H	-3.37029300	2.19788300	0.73142700
H	0.66079900	1.25587500	1.82725900
H	0.98045800	0.09365600	-0.33649700
C	-3.31478400	0.96581700	-1.66824600
H	-3.14868300	0.50881100	-2.63880100
N	-4.55018700	1.57744000	-1.46878100
N	-5.43247800	0.82408500	-0.82946800
C	-6.69095500	1.29671400	-0.56758700
S	-7.16151600	2.88531400	-1.06111500
N	-7.58641400	0.59792300	0.05554600
C	-8.70453900	2.58284400	-0.36572800
C	-8.72797000	1.33811400	0.16517000
H	-9.49359800	3.31485400	-0.38366600
H	-5.20693000	-0.11409500	-0.50839200
H	-1.51766200	2.30605400	2.34982000
H	-0.86489600	-0.02807000	-1.96109200
C	-9.90762400	0.72504200	0.85641000
F	-9.62782100	0.40698000	2.12191200
F	-10.31073500	-0.39198300	0.24794800
F	-10.94239000	1.57289000	0.87229300

Compound 4 S_0 from Figure 4 optimized at DFT/M062X/def2-TZVP

35

-1292.439815 a.u.

C	-0.89892100	0.10852300	-0.47679100
C	-2.07092800	0.83803300	-0.20496900
C	-1.97250600	2.06390500	0.43263000
C	-0.73799100	2.58218500	0.80704000
C	0.41568900	1.85961500	0.53753700
C	0.32984900	0.62509200	-0.10383600
H	-2.86950600	2.63029900	0.64490400
H	1.38970900	2.23437300	0.81562200
H	1.24111400	0.08096900	-0.30320100
C	-3.36761100	0.28619400	-0.60053000
H	-3.37391900	-0.68251300	-1.09956200
N	-4.44784700	0.91934200	-0.36672600
N	-5.60437000	0.36219300	-0.75258800
C	-6.78786400	1.00004100	-0.51890700
S	-6.84222200	2.53619700	0.26824000
N	-7.92502500	0.49179400	-0.87244100
C	-8.56728100	2.49579500	0.10858900
C	-8.93197100	1.34323800	-0.51477200
H	-9.17875500	3.30024700	0.47420300
H	-5.63867900	-0.53857600	-1.21610800
O	-0.76134700	3.79241600	1.42647200
O	-1.06382500	-1.08884900	-1.10580700
C	0.47136900	4.34427300	1.82212700
H	0.24270800	5.29678900	2.29256500
H	0.98294200	3.69845800	2.54181500
H	1.12560100	4.51319100	0.96177600
C	0.09099300	-1.84200700	-1.39286800
H	0.62672500	-2.10849600	-0.47764500
H	-0.25020800	-2.74725000	-1.88785700
H	0.76380600	-1.29537200	-2.05930200
O	-10.17199400	0.93279700	-0.82602100
C	-11.20946400	1.81250200	-0.45583000
H	-12.13881700	1.34670800	-0.77082300
H	-11.22188600	1.96434900	0.62730000
H	-11.09469400	2.77963800	-0.95367000

Compound 4 T_1 from Figure 4 optimized at DFT/M062X/def2-TZVP

35

-1292.362617 a.u.

C	-0.86477200	0.06238000	-0.76834700
C	-2.11595100	0.74645200	-0.79812700
C	-2.25901200	1.90083500	-0.01166000
C	-1.22042800	2.36739100	0.77986400
C	-0.00897300	1.68660600	0.80202400
C	0.15792100	0.53490500	0.02271000
H	-3.18987600	2.45219600	-0.02905000
H	0.81606200	2.02777400	1.40903800
H	1.10916500	0.02425600	0.05573400
C	-3.16852200	0.25635100	-1.59910400
H	-3.01652800	-0.59408500	-2.25167000
N	-4.38602500	0.92579300	-1.69611200
N	-5.27014700	0.57522200	-0.77494600
C	-6.51087300	1.15591100	-0.73161000
S	-6.96183300	2.37585100	-1.85842300
N	-7.39556300	0.82233300	0.15557500
C	-8.49608400	2.45596400	-1.06741700
C	-8.52340500	1.56302100	-0.03692100
H	-9.25938800	3.13473100	-1.40208400
H	-5.05805200	-0.12256400	-0.06670800
O	-1.47687600	3.49624700	1.49535300
O	-0.78519400	-1.04496800	-1.54961000
C	-0.44432100	4.00256300	2.30623000
H	-0.84171300	4.89318600	2.78578500
H	-0.14979300	3.27985200	3.07281600
H	0.43275600	4.27282400	1.71097900
C	0.43376800	-1.75000600	-1.56294500
H	0.68135000	-2.12844200	-0.56734000
H	0.29593800	-2.58560000	-2.24378500
H	1.25049800	-1.11800500	-1.92233500
O	-9.53044900	1.32184200	0.81586100
C	-10.69416700	2.09269600	0.61278400
H	-11.40595800	1.78507700	1.37321800
H	-10.47713200	3.15910300	0.72114000
H	-11.11354700	1.90839900	-0.38034700

Compound 5 S_0 from Figure 4 optimized at DFT/M062X/def2-TZVP

32

-1960.135994 a.u.

C	-0.95619000	0.13259400	-0.57480700
C	-2.14661500	0.77357600	-0.21628300
C	-2.06324500	1.96697200	0.50598200
C	-0.83548300	2.49029300	0.85109800
C	0.34392700	1.84823000	0.49157600
C	0.27684800	0.66780600	-0.22248300
H	-2.97560000	2.47194800	0.78948800
H	1.30102400	2.26971600	0.76905400
H	1.18320400	0.15536900	-0.51014100
C	-3.46454300	0.23247900	-0.57437000
H	-3.52527600	-0.69992000	-1.13211400
N	-4.51995600	0.85733200	-0.22987800
N	-5.70580600	0.34730100	-0.56553100
C	-6.85607900	0.99718600	-0.21161400
S	-6.81621200	2.48512100	0.67484800
N	-8.02903000	0.54590300	-0.51343600
C	-8.53749100	2.49638200	0.63231400
C	-8.97784100	1.40480800	-0.02950800
H	-9.11509400	3.28401300	1.08440600
H	-5.79538500	-0.51860800	-1.08623300
C	-0.73726800	3.77732200	1.62638000
C	-0.98854800	-1.15786400	-1.35443000
C	-10.41926900	1.07285900	-0.26658300
F	-0.07354300	3.60126300	2.77531400
F	-1.93326000	4.28279700	1.92824000
F	-0.07051600	4.70909700	0.93413500
F	0.23187400	-1.62599900	-1.60891200
F	-1.60697500	-1.00923100	-2.53587000
F	-1.64918300	-2.12087100	-0.69357000
F	-10.75238600	-0.09787000	0.28089200
F	-10.70017500	0.99404400	-1.56888300
F	-11.21627300	2.00768800	0.26286900

Compound 5 T_1 from Figure 4 optimized at DFT/M062X/def2-TZVP

32
-1960.058465 a.u.

C	-0.95248900	0.14214800	-0.76425400
C	-2.20980000	0.79693400	-0.70519800
C	-2.35134900	1.84594100	0.23241100
C	-1.30609800	2.20197300	1.05138100
C	-0.07967100	1.54533200	0.98442500
C	0.08377000	0.51527900	0.06951700
H	-3.29107100	2.37743400	0.28744200
H	0.73248100	1.83676800	1.63644700
H	1.02963700	-0.00324500	0.00611800
C	-3.30187100	0.44093200	-1.53334600
H	-3.20435900	-0.29761900	-2.31974200
N	-4.50453800	1.13201700	-1.48155300
N	-5.40691300	0.62195900	-0.65993800
C	-6.64119400	1.20795900	-0.53022900
S	-7.05019700	2.63224600	-1.41739300
N	-7.55392300	0.73551200	0.25664500
C	-8.59228700	2.59110900	-0.66067900
C	-8.66147000	1.52898500	0.17584100
H	-9.34869000	3.32929100	-0.86538200
H	-5.22733900	-0.21320200	-0.10826500
C	-1.45615000	3.32535700	2.04236800
C	-0.73860600	-0.98515200	-1.73760100
C	-9.85595800	1.16966800	1.00731200
F	-1.19777200	2.91000300	3.28827000
F	-2.68412300	3.84606500	2.04139000
F	-0.59926800	4.31994700	1.78135000
F	0.48732300	-1.50143800	-1.65266900
F	-0.91028500	-0.58877300	-3.00739900
F	-1.60278600	-1.99150200	-1.53000900
F	-9.56714400	1.16995000	2.30936900
F	-10.31927900	-0.04380600	0.70377800
F	-10.84894900	2.04366300	0.81154100

Compound HM610 S_0 from Figure 4 optimized at DFT/M062X/def2-TZVP

34

-1515.005122 a.u.

C	-0.92744300	0.24878100	-0.70986300
C	-2.08491500	0.93204400	-0.29427000
C	-1.95684500	2.11709500	0.41177800
C	-0.70540700	2.64063800	0.71520900
C	0.43441200	1.96377900	0.30349400
C	0.31858300	0.77077200	-0.40721600
H	-2.84285300	2.64738000	0.73448400
H	1.42121600	2.34392100	0.52319100
H	1.21998900	0.26280100	-0.71636000
C	-3.39896100	0.37483000	-0.61675700
H	-3.42716400	-0.56338000	-1.17032000
N	-4.46883700	0.96717200	-0.26157200
N	-5.64246200	0.40001400	-0.58914600
C	-6.81139300	0.99765600	-0.22947900
S	-6.82007500	2.49457000	0.64792500
N	-7.97280900	0.49943200	-0.51266200
C	-8.54237300	2.43806700	0.62745000
C	-8.94642900	1.32528100	-0.02147200
H	-9.14596500	3.20470900	1.08129400
H	-5.69433800	-0.47359200	-1.10061400
C	-10.37601400	0.93631800	-0.23686800
F	-10.65933100	-0.24263800	0.32309900
F	-10.67323800	0.83569500	-1.53496800
F	-11.20433400	1.84295400	0.29572400
O	-0.69894400	3.80771300	1.41145700
O	-1.12276100	-0.90909000	-1.39937300
C	0.55247400	4.36557900	1.73559600
H	0.34587200	5.28114300	2.28306000
H	1.13665900	3.68993200	2.36703700
H	1.12618200	4.60351600	0.83518800
C	0.01756700	-1.61683000	-1.82846800
H	0.63176700	-1.92775400	-0.97912000
H	-0.34826500	-2.49657000	-2.35085100
H	0.62233700	-1.01408200	-2.51129400

Compound HM610 T_1 from Figure 4 optimized at DFT/M062X/def2-TZVP

34

-1514.927018 a.u.

C	-0.91335400	0.26495000	-1.09128000
C	-2.17345500	0.91758800	-0.94773600
C	-2.27757200	1.96180500	-0.01447600
C	-1.19262200	2.35143500	0.75551500
C	0.02765300	1.70176200	0.60739500
C	0.15611600	0.66051000	-0.31971600
H	-3.21512700	2.48898600	0.10314000
H	0.88872000	1.98471700	1.19395300
H	1.11487000	0.17267900	-0.41709000
C	-3.27121200	0.50386500	-1.72949200
H	-3.15499400	-0.25649300	-2.49123000
N	-4.50331900	1.15325800	-1.66268400
N	-5.31780000	0.67092500	-0.73555000
C	-6.56194600	1.21223100	-0.55479400
S	-7.09509800	2.54317900	-1.52136900
N	-7.39748000	0.77419800	0.33358200
C	-8.57762900	2.50827400	-0.65110700
C	-8.54026200	1.51801700	0.27067900
H	-9.37854000	3.19737800	-0.85690400
H	-5.04661800	-0.09926800	-0.12964000
O	-1.41425700	3.37547200	1.62265300
O	-0.87356200	-0.73364100	-2.00879000
C	-0.33422400	3.80277600	2.41812500
H	-0.71094500	4.61648400	3.03203600
H	0.02554500	2.99782800	3.06547500
H	0.49315000	4.16690600	1.80224500
C	0.35221300	-1.40259700	-2.19589100
H	0.67579400	-1.89640600	-1.27561100
H	0.17801900	-2.14939100	-2.96561500
H	1.13099900	-0.71105200	-2.52842100
C	-9.65585000	1.18599600	1.21389900
F	-10.69960200	2.00097300	1.02165800
F	-10.08733400	-0.06613800	1.04806000
F	-9.27937300	1.30807500	2.488478000
